# Identification of renal cyst cells of type I Nephronophthisis by single-nucleus RNA sequencing

**DOI:** 10.3389/fcell.2023.1192935

**Published:** 2023-07-31

**Authors:** Qianying Wang, Baojuan Zou, Xiaoya Wei, Hongrong Lin, Changmiao Pang, Lei Wang, Jinglin Zhong, Huamu Chen, Xuefei Gao, Min Li, Albert C. M. Ong, Zhihui Yue, Liangzhong Sun

**Affiliations:** ^1^ Department of Pediatrics, Nanfang Hospital, Southern Medical University, Guangzhou, China; ^2^ State Key Laboratory of Respiratory Disease, National Clinical Research Center for Respiratory Disease, Guangzhou Institute of Respiratory Health, The First Affiliated Hospital of Guangzhou Medical University, Guangzhou, China; ^3^ Department of Physiology, School of Basic Medical Sciences, Southern Medical University, Guangzhou, China; ^4^ Kidney Genetics Group, Academic Nephrology Unit, Department of Infection, Immunity and Cardiovascular Disease, University of Sheffield Medical School, Sheffield, United Kingdom; ^5^ Department of Pediatrics, The First Affiliated Hospital, Sun Yat-sen University, Guangzhou, China

**Keywords:** renal cyst, single-nucleus RNA sequencing, Nephronophthisis, NPHP1, Niban1

## Abstract

**Background:** Nephronophthisis (NPH) is the most common genetic cause of end-stage renal disease (ESRD) in childhood, and *NPHP1* is the major pathogenic gene. Cyst formation at the corticomedullary junction is a pathological feature of NPH, but the mechanism underlying cystogenesis is not well understood. The isolation and identification of cystic cell subpopulation could help to identify their origins and provide vital clues to the mechanisms underlying cystogenesis in NPH.

**Methods:** Single-nucleus RNA sequencing (snRNA-seq) was performed to produce an atlas of NPHP1 renal cells. Kidney samples were collected from WT (*Nphp1* ^+/+^) mice and NPHP1 (*Nphp1* ^del2-20/del2-20^) model mice.

**Results:** A comprehensive atlas of the renal cellular landscape in NPHP1 was generated, consisting of 14 basic renal cell types as well as a subpopulation of DCT cells that was overrepresented in NPHP1 kidneys compared to WT kidneys. GO analysis revealed significant downregulation of genes associated with tubular development and kidney morphogenesis in this subpopulation. Furthermore, the reconstruction of differentiation trajectories of individual cells within this subpopulation confirmed that a specific group of cells in *NPHP1* mice become arrested at an early stage of differentiation and proliferate to form cysts. We demonstrate that *Niban1* is a specific molecular marker of cystic cells in both mice and human NPHP1.

**Conclusion:** In summary, we report a novel subpopulation of DCT cells, marked by *Niban1,* that are classified as cystic cells in the NPHP1 mice kidney. These results offer fresh insights into the cellular and molecular basis of cystogenesis in NPH.

## 1 Introduction

Nephronophthisis (NPH) is an autosomal recessive genetic cystic kidney disease (Wolf and Hildebrandt, 2011) that is the most common genetic cause of renal failure in childhood and accounts for 5%–15% of children with end-stage renal disease (ESRD) (Oud et al., 2017). NPH patients usually reach ESRD at teenagers. Renal cyst formation at the corticomedullary junction and diffuse interstitial fibrosis with mononuclear cell infiltration are key pathological characteristics of NPH. However, the mechanism of renal cystogenesis is still not well understood in NPH nor in other renal ciliopathies, such as autosomal dominant polycystic kidney disease (ADPKD).

Pathogenic variants in NPH genes and those of other renal ciliopathies affect proteins related to the structure or function of primary cilia (McConnachie et al., 2021). To date, more than 20 genes linked to NPH have been identified, of which *NPHP1* accounts for 50%–60% (König et al., 2017; Srivastava et al., 2017). These gene mutations were associated with abnormalities in ciliary signaling pathways, including the cAMP, Notch3, Sonic Hedgehog (Shh), WNT and Hippo pathways (Goetz and Anderson, 2010; Calvet, 2015; Elbediwy and Thompson, 2018; Idowu et al., 2018; Müller and Schermer, 2020), which in turn result in changes in cell polarity, proliferation and apoptosis, which predispose renal cells to cyst formation (Wilson et al., 1992; Lanoix et al., 1996). The isolation and identification of cyst cell subpopulation would greatly help to elucidate the mechanism of renal cystogenesis in ciliopathies. However, this has been technically challenging to achieve by conventional isolation methods (Pei, 2001). One recent study identified the origin of cyst cells by direct excision of cysts from ADPKD patients for single-cell RNA sequencing (scRNA-seq) (Li et al., 2021b). However, there are no relevant studies in NPH.

Single-cell/nucleus RNA sequencing (sc/nRNA-seq) provides informations about the transcriptomes of individual cells and assesses differences in gene expression between individual cells. Sc/nRNA-seq analysis can reveal changes in cell types and gene expression in disease states, as well as biomarkers and activation of intracellular signaling pathways in the early stages of disease (Wilson et al., 2019; Paik et al., 2020). The application of sc/nRNA-seq can also help to identify rare cell populations based on different gene expression pattern between individual cells and has been used successfully to identify pathogenic cell populations in various diseases, including kidney diseases (Karaiskos et al., 2018; Liao et al., 2020; Li et al., 2021b; Lu et al., 2021).

We recently reported the generation and characterization of a new NPHP1 (*Nphp1* ^*del2-20/del2-20*^) mouse model with a kidney phenotype resembling that of human NPH(Li et al., 2021a). In the present study, single-nucleus RNA sequencing (snRNA-seq) was performed to study the composition of renal cell types and the molecular signature of cyst lining cells in these mice. Our results revealed a comprehensive cellular atlas of the NPHP1 mice kidney for the first time and identified a novel subpopulation of NPHP1 renal cyst cells defined by a specific molecular marker.

## 2 Materials and methods

### 2.1 Laboratory animals

C57BL/6J mice were bred by GemPharmatech Co., Ltd. and housed at the animal facility of Nanfang Hospital Southern Medical University on a 12 h/12 h light/dark cycle at 21°C and 50%–55% humidity under specific pathogen-free (SPF) conditions with food and water available *ad libitum*. Mice were anesthetized with 3% sodium pentobarbital and sacrificed at 12 or 36 weeks of age.

### 2.2 Single-nucleus isolation and library preparation

From fresh-frozen mice kidney tissue, approximately 100 mg was cut into < 2-mm pieces and homogenized using a Dounce homogenizer in ice-cold homogenization buffer (0.25 M sucrose) (Sigma‒Aldrich RNase and DNase free, ultrapure grade), 5 mM CaCl_2_, 3 mM MgAc_2_, 10 mM Tris-HCl pH 8.0, 0.1 mM EDTA, 1x protease inhibitor (Thermo Scientific, 78425), and 1 U/μL RiboLock RNase inhibitors (Thermo Scientific E, O0381), 15–20 strokes with the A pestle, followed by 5–10 strokes of the B pestle. Homogenates were passed through a 70-μM cell strainer to collect the nuclear fraction. The nuclear fraction was mixed with an equal volume of 50% iodixanol to a final concentration of 25%. Then, 33% and 30% iodixanol solution and the 25% iodixanol solution containing the nuclei were added to the centrifuge tube and centrifuged at 3,234 × g for 20 min at 4°C. The nuclei were collected from the 30%–33% iodixanol interface. The nuclei were resuspended in nuclear wash and resuspension buffer (0.04% bovine serum albumin, 0.2 U/μL RiboLock RNase inhibitors, 500 mM mannitol and 0.1 mM PMSF protease inhibitor in phosphate-buffered saline) and pelleted for 5 min at 500 × g and 4°C. The nuclei were passed through a 40 μM cell strainer to remove cell debris and large clumps. After another round of centrifugation and resuspension, the nuclei concentration was manually determined using trypan blue counter staining and a hemocytometer and then adjusted to 700–1,200 nuclei/μL. The 10× Genomics GemCode Single-cell instrument was used to generate high-quality cDNA libraries.

### 2.3 Library quality control and sequencing

The DNA 1000 Assay Kit (Agilent Technologies) was used for library quality control. The concentration of cDNA samples was measured using Qubit 3.0 with a concentration guide range of 0.2–1,000 ng/μL. Finally, the ABI StepOnePlus Real-Time PCR System (Life Technologies) was used for quantification and pooling. The Single Cell 3’ Protocol produced Illumina-ready sequencing libraries. A Single Cell 3’ Library comprised standard Illumina paired-end constructs that begin and end with P5 and P7. The Single Cell 3’ 16 bp 10x Barcode and 10 bp UMI were encoded in Read 1, while Read 2 was used to sequence the cDNA fragment. Sample index sequences were incorporated as the i7 index read. Read 1 and Read 2 were standard Illumina ^®^ sequencing primer sites used in paired-end sequencing.

### 2.4 Human kidney tissue procurement

The kidney tissue of 3 NPH patients (F2-P52, F47-P53, F49-P56) (Yue et al., 2020) and 3 control patients was obtained from kidney biopsies. Control patient 1: female, 11 years old, diagnosed with purpura nephritis at stage IIIa. Control patient 2: male, 12 years old, diagnosed with primary nephrotic syndrome with minimal change nephropathy. Control patient 3: male, 11 years old, diagnosed with primary nephrotic syndrome with minimal change nephropathy. The kidney tissue of NPH patients and control patients were fixed stained in the same conditions (See Supplementary Material S1 for details).

### 2.5 Statistics

Experiments were repeated independently at least three times. Data were shown as the mean ± standard deviation (SD). Student’s *t*-test was performed to compare groups. *p* < 0.05 was considered statistically significant. Statistical analysis was performed using GraphPad Prism 8.0.

### 2.6 Supplementary methods

Details of the bioinformatic analysis of single-nucleus RNA sequencing data, kidney histopathology, immunohistochemistry and immunofluorescence studies, as well as TUNEL assays for apoptosis were provided in the Supplementary Material S1.

## 3 Results

### 3.1 Construction of a cellular atlas for the NPHP1 kidney

**FIGURE 1:**
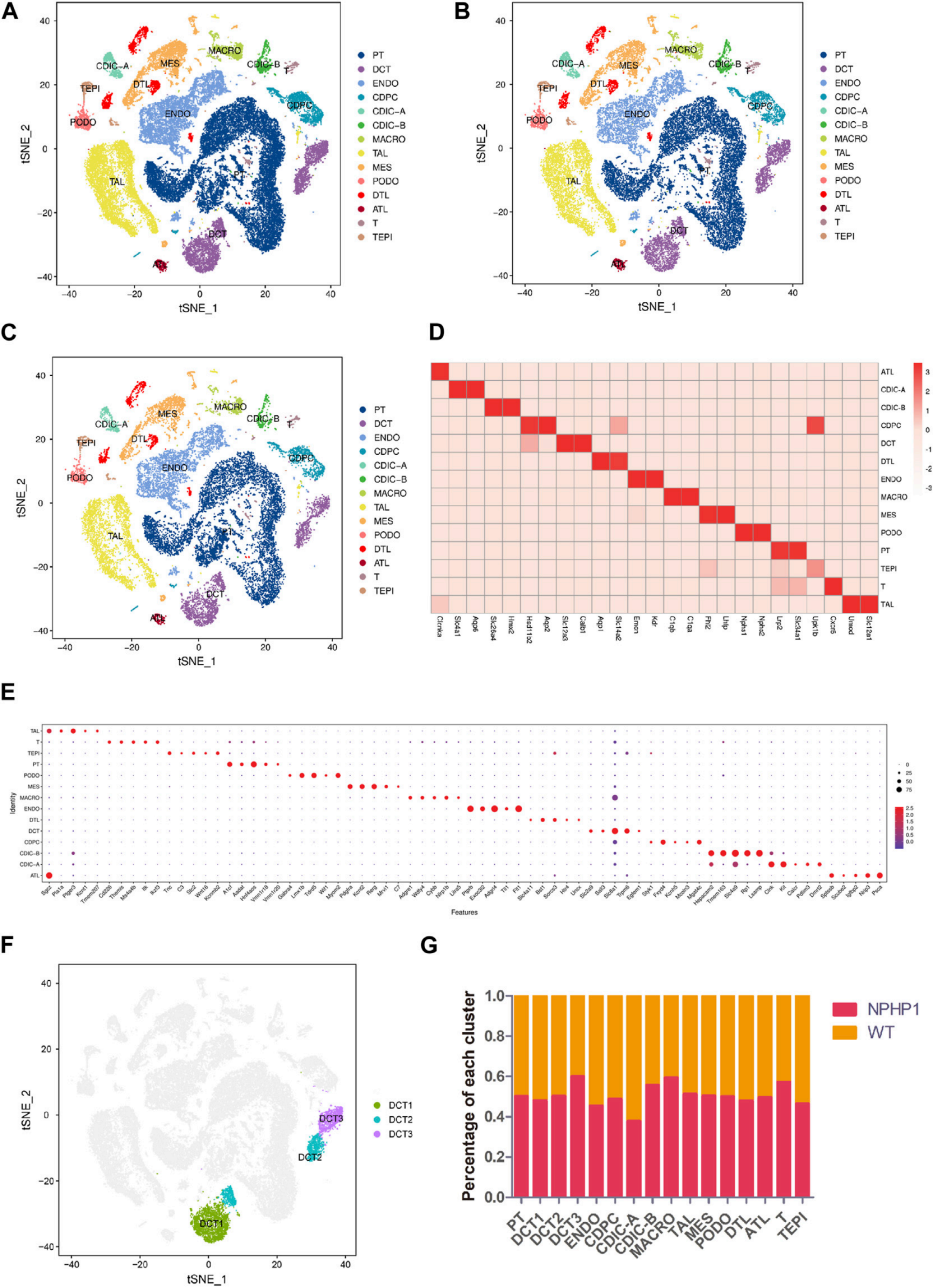
Classification of kidney cells of NPHP1 and wild-type mice by snRNA-seq. **(A)** t-SNE projection of renal cells analyzed by snRNA-seq. Cells are colored according to distinct cell populations, as indicated. PT, proximal tubule cells; ENDO, endothelial cells; TAL, thick ascending limbs of the loops of Henle; DCT, distal convoluted tubule cells; MES, mesangial cells; CD-PC, collecting duct principal cells; CD-IC, collecting duct intercalated cells; MACRO, macrophages; PODO, podocytes; DTL, descending thin limbs of the loops of Henle; ATL, ascending thin limbs of the loops of Henle; T, T lymphocytes; TEPI, transitional epithelium. **(B,C)**: t-SNE projection of NPHP1 **(B)** and WT **(C)** respectively. **(D)** Heatmap of the relative expression of canonical cell markers in each type of cell population. **(E)** Top 5 distinct genes for each cell population (excluding the canonical cell markers shown above). The color and size of the dots indicate the relative average expression level and the proportion of cells expressing the gene, respectively. **(F)** Subclusters of cluster DCT (divided into DCT1, DCT2 and DCT3). **(G)** Percentage of each cluster in NPHP1 and WT samples. Data calculated from (number of cells of each cluster in NPHP1/total number of cells of NPHP1)/(number of cells of each cluster in WT/total number of cells of WT). The bar chart shows that the DCT3 and macrophage clusters were significantly more represented in NPHP1 than in the control samples by a factor of approximately 1.46–1.49; T lymphocytes were overrepresented by a factor of 1.33, and the remaining cell types differed by factors of less than 1.30.

We performed snRNA-seq on kidney samples from adult WT (wild-type) and NPHP1 mice (12 weeks old), the latter of which had preexisting renal cyst formation. We isolated and sequenced 51556 nuclei from whole-kidney nucleus suspensions from 2 pairs of NPHP1 and WT mice. After applying stringent quality controls, we retained 48116 nuclei, including 27376 from NPHP1 mice and 20740 from WT mice, for further analysis. The median numbers of genes per nucleus were 1,623 (NPHP1) and 1,578 (WT). Clustering analysis identified 14 distinct cell clusters (Figures 1A–C). We generated cluster-specific marker genes by performing upregulated gene expression analysis and according to the known cell type-specific markers (Figure 1D) to identify each cluster, which were proximal tubule cell (PT), endothelial cell (ENDO), thick ascending limbs of the loops of Henle (TAL), distal convoluted tubule cell (DCT), mesangial cell (MES), collecting duct principal cell (CDPC), collecting duct intercalated cell (type A and B) (CDIC-A/B), macrophage (MACRO), podocyte (PODO), descending thin limbs of the loops of Henle (DTL), ascending thin limbs of the loops of Henle (ATL), T lymphocyte (T) and transitional epithelium (TEPI). Apart from these known markers, we also found additional markers for each cell type (Figure 1E). Further analysis also identified three subclusters of DCT cells, denoted as DCT1, DCT2 and DCT3 (Figure 1F). Notably, DCT3 cells and macrophages were significantly overrepresented in NPHP1 mice relative to wild-type mice (Figure 1G). Compared with snRNA-seq performed on adult mouse kidneys (Lu et al., 2021), in which 16 cell populations were analyzed and identified, we have also identified PT, DCT1/2, CDPC, CDIC-A/B, MES, PODO, ENDO, TAL, DTL, ALT, transitional epithelium and immune cells. We did not obtain CNT, juxtaglomerular cells, proliferative cells, fibroblasts as well as some new subclusters of PT as shown in their study. However, we identified a new subpopulation of DCT, which were not previously reported in adult mouse kidneys studies. The differences in the cell types identified between different studies may related to the different mouse models, varied RNA expression levels and the different thresholds set.

### 3.2 Altered gene expression in each cell type in NPHP1 kidney

**FIGURE 2:**
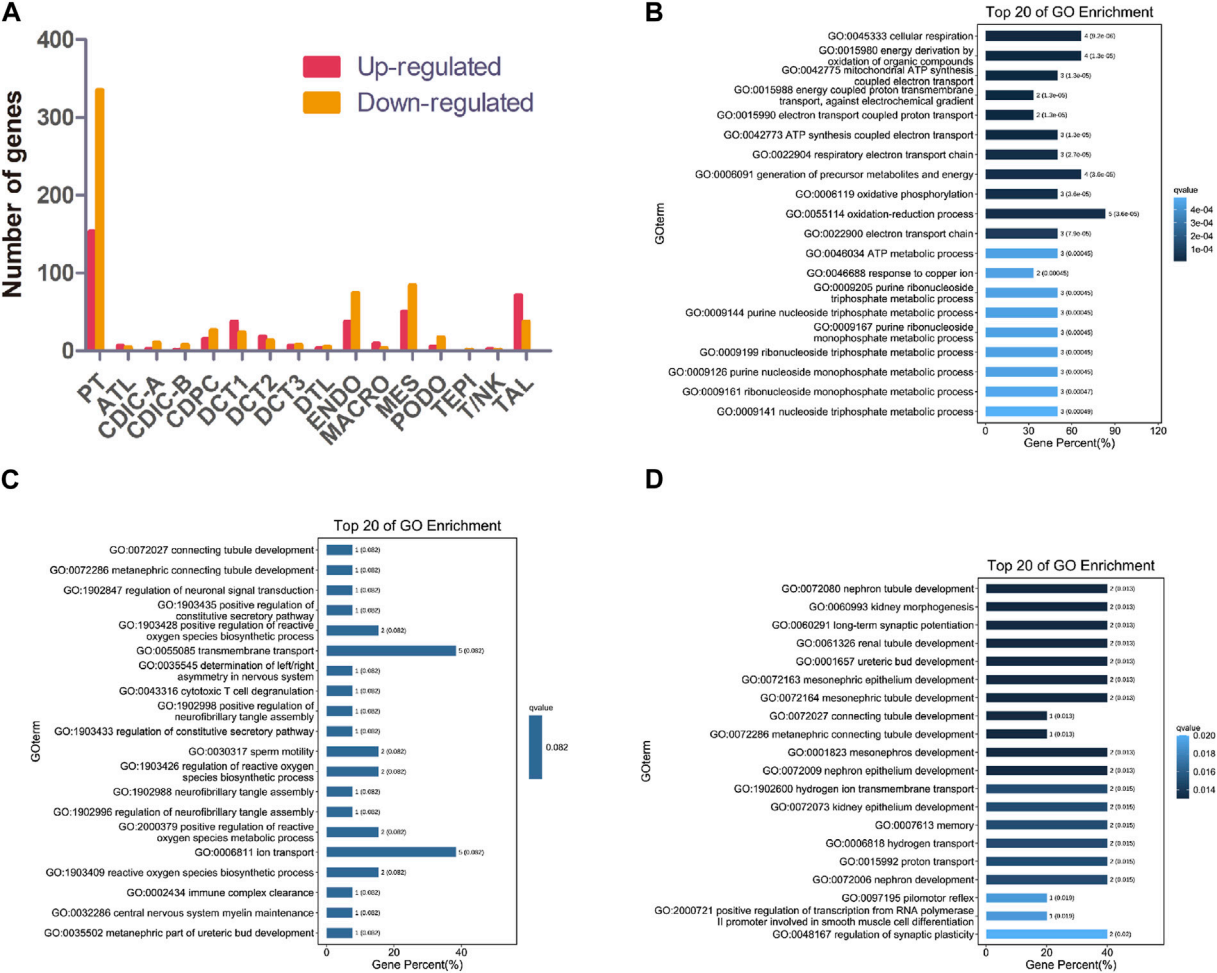
Gene expression changes in different clusters of renal cells between NPHP1 and WT mice. **(A)** The number of up- and downregulated genes in different renal cell clusters in NPHP1 mice relative to control cohorts. **(B)** Bar plot of GO terms for genes upregulated in macrophages. **(C,D)**: Bar plot of GO terms for genes downregulated in DCT2 **(C)** and DCT3 **(D)**.

Knockout of *NPHP1* induced gene expression changes in all renal cell populations (Figure 2A). Gene Ontology (GO) analysis of the upregulated genes in each cluster showed that they were associated with metabolic processes (PT, DCT1, TAL), regulation of molecular function (DCT2), protein ubiquitination (DCT3), regulation of bile acid metabolic process (CDIC-A), negative regulation of biological process (CDPC), regulation of metanephros/kidney size (ATL), regulation of TRAIL-activated apoptotic signaling pathway (DTL), sodium ion export (ENDO), nucleotide catabolic process and signal transduction (MES), regulation of TORC2 signaling and microtubule motor activity (PODO), and mitochondrial ATP synthesis and energy transportation (MACRO). GO analysis of the downregulated genes in each cluster showed that they were related to ion transmembrane transport (PT), lipid biosynthetic process (DCT1), connecting tubule development (DCT2), renal tubule development and kidney morphogenesis (DCT3), regulation of ion transport and metabolic process (CDIC-A), melanosome localization (CDIC-B), regulation of intrinsic apoptotic signaling pathway in response to DNA damage (CDPC), fatty acid biosynthetic process (ATL), chromosome assembly (DTL), cell-substrate adhesion (TAL), regulation of cell motility and biological process (ENDO), developmental process (MES), metabolic process (PODO), regulation of mitochondrial membrane permeability (TEPI), and regulation of antigen processing and presentation (MACRO). GO analysis of the upregulated genes of MACRO identified associations with mitochondrial ATP synthesis as well as energy transportation (Figure 2B). Together with the increased percentage of MACRO in NPHP1 cells (Figure 1G), these data suggested that macrophages were activated in NPHP1. Most importantly, the genes downregulated in DCT2 and DCT3, such as *Calb1*, *Npnt* and *Shank2*, were associated with tubule development and kidney morphogenesis. Suggesting that tubule morphogenesis was blocked in DCT2 and DCT3 (Figures 2C, D). Specifically, DCT2 and DCT3 localized to the corticomedullary junction, where cysts originate. Taken together with the results in Figure 1G showing an increased percentage of cells in the DCT3 cluster, we speculated that cyst cells may originate from the DCT cell population. The other GO process maps were showed in the Supplementary Material S2, S3.

### 3.3 Identification of a cystic subpopulation in NPHP1 kidney

**FIGURE 3:**
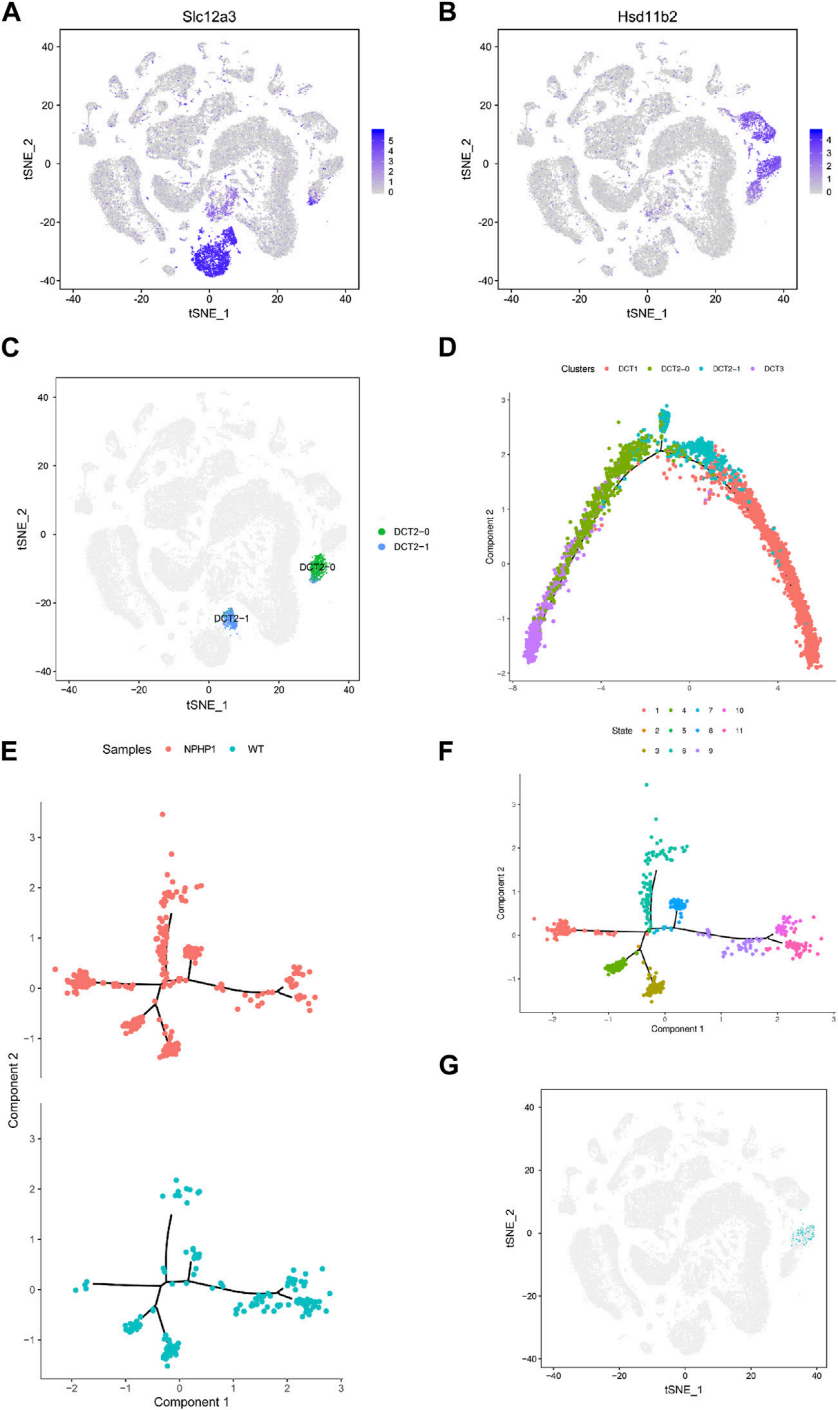
Identification of NPHP1 renal cyst cells. **(A,B)**: The t-SNE projections of the expression levels of the distal convoluted tubule cell marker gene *Slc12a3* **(A)** and of the collecting duct principal cell marker gene *Hsd11b2* **(B)**. The plots are colored according to mean gene expression (blue = high, gray = low). **(C)** DCT2 cells can be further subdivided into two small subpopulations, DCT2-0 and DCT2-1. **(D)** Sorting of single cells along cellular transition trajectories using Monocle2. The subpopulations of DCT1, DCT2 (DCT2-0 and DCT2-1), and DCT3 cells are differentiated along the proposed differentiation axis, and the distribution of different clusters of cells in the cell trajectory, where the DCT3 cells (purple) are located at the differentiation initiation site, which cells are in the early differentiation state, DCT1 cells (red) are located at the end of the differentiation axis, which cells are in a mature state of differentiation, while DCT2 cells are in between. **(E,F)**: Ordering single cells along a cell conversion trajectory using Monocle2. DCT2-0 and DCT3 cells are merged for plotting in low-dimensional space, and samples are shown with different colors **(E)**. The distribution of cells of different differentiation states in the cell track; different colors represent different cell states, there are total of 11 cell states. **(F)**. **(G)** Location mapping of cyst cells on t-SNE plots based on single-cell trajectory isolation. As shown in the figure, cyst cells projecting in the DCT3 cell subpopulation.

Analysis of the gene expression profiles of the three different subpopulations of DCT showed that the gene expression in DCT1 cells was similar to that in normal distal tubule cells, while DCT3 cells had a different gene expression profile. Nonetheless, these cells continued to express some of the marker genes of principal cells. Our single-cell profiling also identified another cell cluster, DCT2, which expressed markers common to DCT1 and DCT3 (Figures 3A, B). It seems likely that the DCT2 subpopulation was a transitional cell type between DCT1 and DCT3, and this cluster could be further subdivided into DCT2-0 and DCT2-1 (Figure 3C). We utilized the Monocle2 analysis toolkit to perform cell trajectory analysis for pseudotime reconstitution of DCT1-3, which confirmed our hypothesis. We found that DCT1-3 cells were differentiated in the same direction, with DCT3 cells at the beginning of the differentiation axis at the initial stage of differentiation, DCT1 cells at the end of the differentiation axis at the mature stage of differentiation, and DCT2 cells (containing DCT2-0 and DCT2-1) located between DCT1 and DCT3 (Figure 3D). Frequency analysis of the DCT subpopulations further revealed that NPHP1 mice contained a higher percentage of DCT3 cells (1.46), while DCT1 and DCT2 were equally represented in both samples (Figure 1G). Since DCT3 cells appear to have downregulated expression of key genes implicated in renal tubular development and kidney morphogenesis, it was plausible that all or some of the cells in DCT3 may not be able to differentiate into normal mature tubular cells and could instead proliferate (see below), resulting in cyst formation. The cell trajectory analysis and the gene expression characteristics of each subpopulation of cells showed that DCT2-0 was similar to DCT3. Therefore, the DCT2-0 and DCT3 cell subsets could both be specific cell subsets associated with the development of renal cysts.

Our target cell populations, DCT2-0 and DCT3, are therefore at a relatively early stage of differentiation. As DCT2-0 and DCT3 cells were present in both the model and control groups, we repeated the cell trajectory analysis using pseudotime reconstitution of both DCT2-0 and DCT3 (Figures 3E, F). As shown, both DCT2-0 and DCT3 cells differentiated along the same axis and branches in both samples, with 5 branching nodes and 11 differentiation states. Comparing the differentiation trajectories, state 1 showed the most significant differences between the two samples, with a cell count of 95/335 in the NPHP1 group compared to 4/176 in the WT group. The temporal differentiation axis shows that state 1 cells are at an earlier stage of differentiation. We mapped state 1 cells on t-SNE plots and found that since they originated from the DCT3 cell population (Figure 3G), they were also strong candidates to be NPHP1 cyst cells. As the Monocle software is susceptible to batch effects, the above analysis of the divergence trajectories for DCT2-0 and DCT3 only shows the data analysis for one pair of batch samples, but similar results could be replicated for another batch.

### 3.4 Niban1 is specifically expressed in renal cyst cells and dilated tubule cells of NPHP1 mice

Next, we sought to identify a specific marker of the cyst cell populations and validate their localization in cysts. Since the cystic cells originated from DCT3 cells, we screened for genes upregulated in cystic cells whose expression differed significantly from the rest of the DCT3 subpopulation (Supplementary Table S1). First, of the genes that were preferentially expressed in cystic cells but not altered or significantly underexpressed in other renal cells, only *Niban1* met our criteria. Next, we selected genes that were significantly upregulated in cystic cells but not altered in other DCT cell populations and identified 10 genes based on the tSNE plots: *Prr5l, Niban1, Plcd3, Aqp3, Col26a1, Fanca, Sgcd, Hdac9, Pde1c* and *Kcnc2* (Supplementary Table S2)*.* Together with analysis using the gene expression map and difference multiplier, the mRNA levels of genes *Prr5l*, *Hdac9* and *Kcnc2* were initially screened to be significantly upregulated in cystic cells and either underexpressed or absent in other cell populations. We also included *Niban1* because of the large difference in its expression between cystic cells and the rest of the DCT3 subpopulation. At the protein level, of Niban1, Prr5l, Hdac9 and Kcnc2, only Niban1 and Hdac9 were specifically expressed in cystic cells, and Niban1 was more significantly elevated.

**FIGURE 4:**
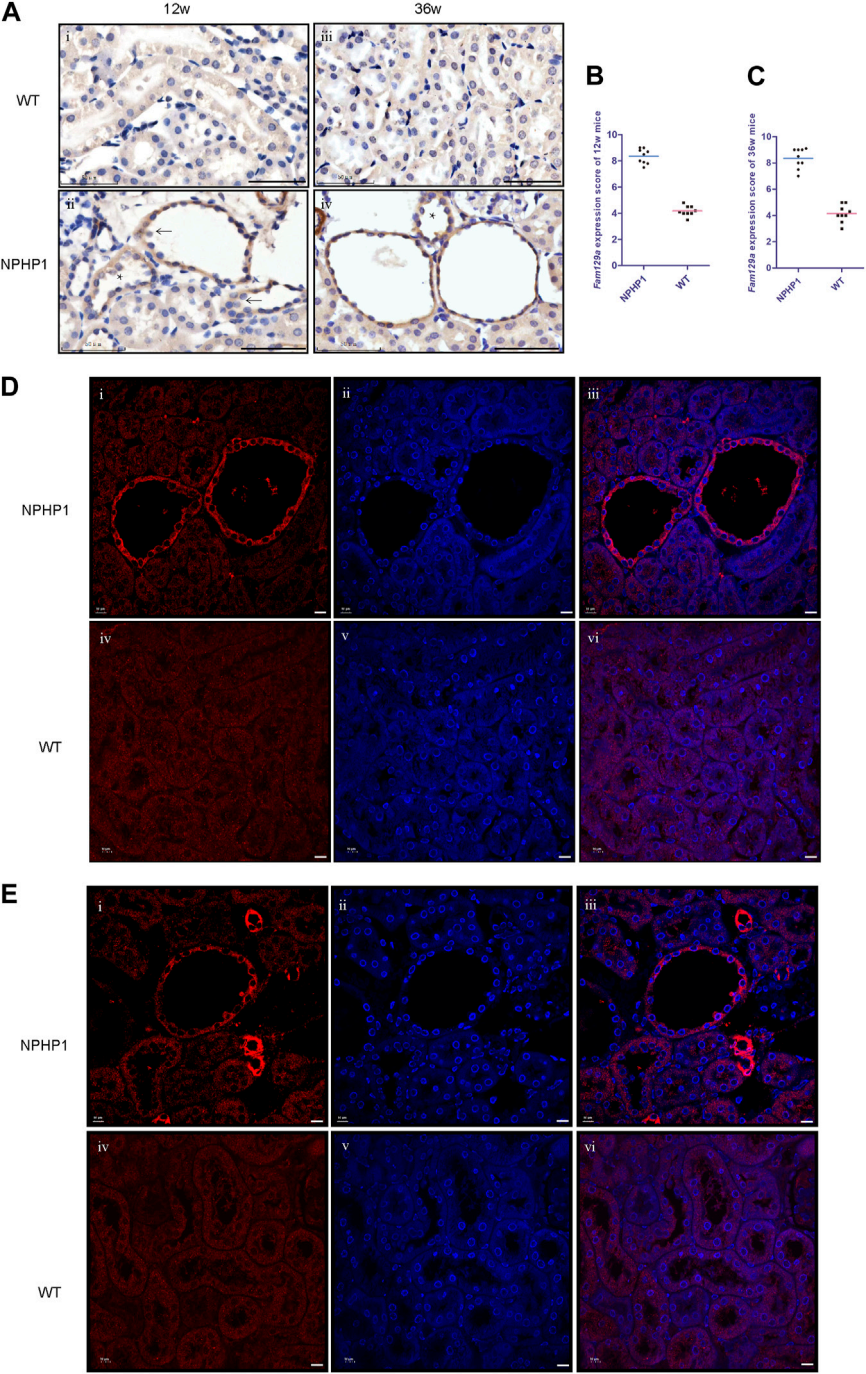
Immunohistochemistry and immunofluorescence staining of Niban1 in kidney tissue of NPHP1 and WT mice. **(A)** Cystic cells and dilated tubular cells of NPHP1 mice were positively stained (brown, ii and iv), whereas uninvolved tubule cells of NPHP1 mice (ii and iv) and normal tubule cells of WT mice (i and iii) were negative. (Immunohistochemistry; i and ii, 12 w; iii and iv, 36 w; arrows point to cyst cells, * point to dilated tubule; Scale bar: 50 μm). **(B,C)**: Niban1 staining score in 12 w (B, *p* < 0.05) and 36 w (C, *p* < 0.0001) mouse kidneys was quantified. *n* = 6 of each group. **(D,E)**: NPHP1 (i–iii) and WT (iv–vi) mice at 12 w **(D)** and 36 w **(E)**. Nuclei were stained with DAPI (blue) and Niban1 (red). (Immunofluorescence, scale bar: 10 μm). (*As our specimen was not perfused, some vascular enhancement is present in Figures E i-iii.*).

*Niban1* (HGNC:16784, localized on chromosome 1q25.3), encodes a protein that is mainly expressed in the cytoplasm and may play a role in the regulation of cell proliferation and death. Both immunohistochemistry and immunofluorescence staining showed that Niban1 was highly expressed in renal cystic cells in NPHP1 mice kidney at 12 and 36 weeks, moderately expressed in dilated tubular cells, but absent from uninvolved tubular cells of NPHP1 mice and normal tubular cells of WT mice (Figure 4). Co-staining of Tamm-Horsfall protein (a glycoprotein secreted by distal convoluted tubule epithelial cells) and Niban1 confirmed that the cystic cells were indeed derived from distal convoluted tubule (Supplementary Figure S1).

### 3.5 Niban1 is specifically expressed in renal cystic cells in children with NPHP1

**FIGURE 5:**
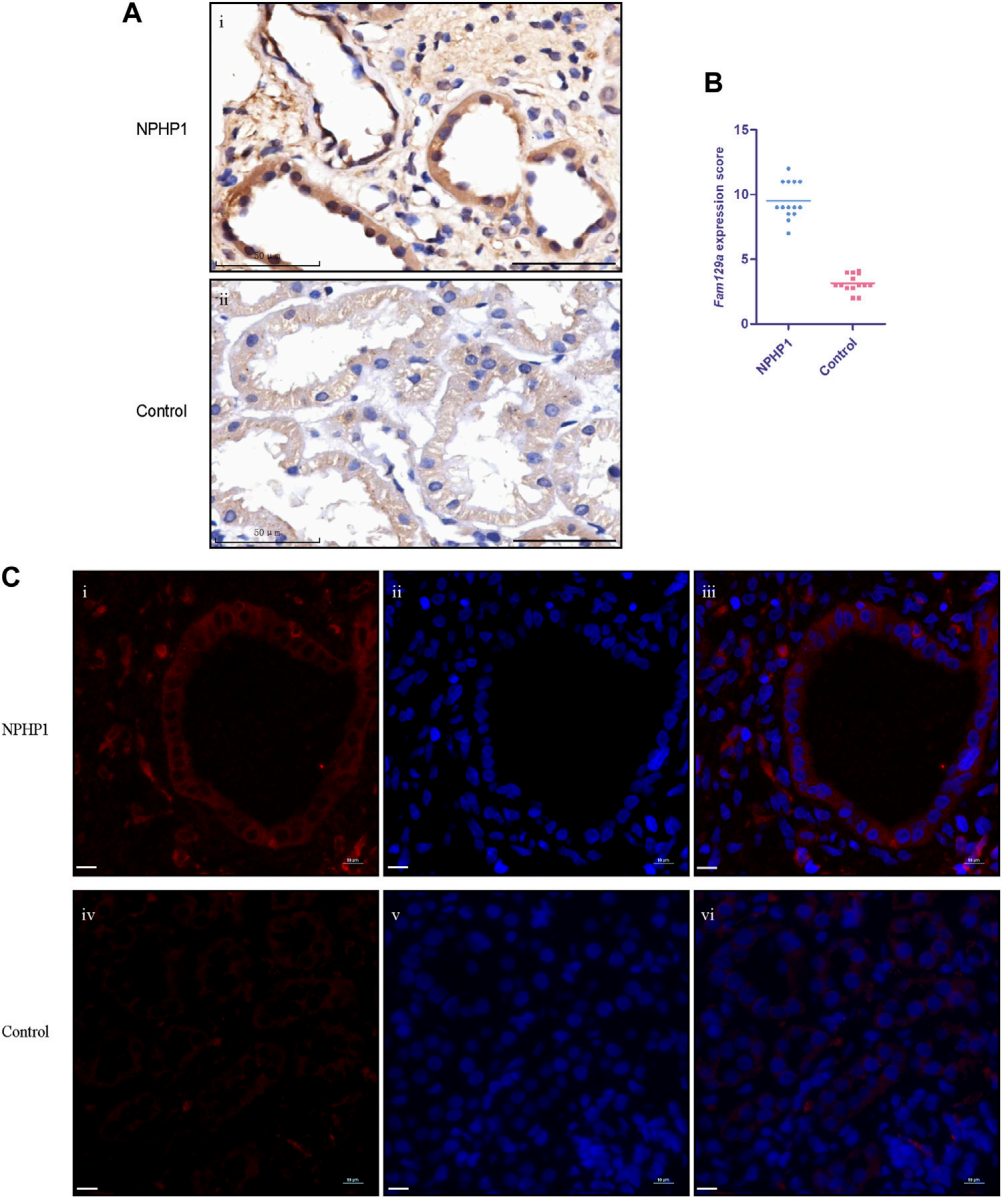
Immunohistochemistry and immunofluorescence staining for Niban1 in kidney tissue of NPHP1 patients and controls. **(A)** The expression of Niban1 was confined to cystic cells and dilated tubular cells (brown). (Immunohistochemistry; i, NPHP1 patients; ii, control patients; Scale bar: 50 μm). **(B)** The scater dot plot shows the kidney tissue Niban1 staining scores of NPHP1 patients and controls (number of fields of view, *n* = 14 of each group. *p* < 0.001). **(C)** The expression of Niban1 was mainly confined to cystic cells and dilated tubular cells in NPHP1 patients [immunofluorescence; NPHP1 (i–iii) and control (iv–vi) patients. Nuclei were stained with DAPI (blue) and Niban1 (red). Scale bar: 10 μm].

To study whether *Niban1* is also expressed in the renal cyst cells of NPHP1 patients, we performed immunohistochemistry and immunofluorescence on paraffin-embedded specimens of kidney biopsy tissue from 3 NPHP1 patients and 3 control patients. Similar to the findings in NPHP1 mice, *Niban1* expression was confined to the renal cyst cells and dilated tubular cells of NPHP1 patients and absent from the uninvolved tubular cells of NPHP1 patients and normal tubular cells of control patients (Figure 5). We concluded that *Niban1* is a specific molecular marker of NPHP1 patient renal cyst cells.

### 3.6 Enhanced proliferative and apoptotic activity in NPHP1 renal cyst cells

**FIGURE 6:**
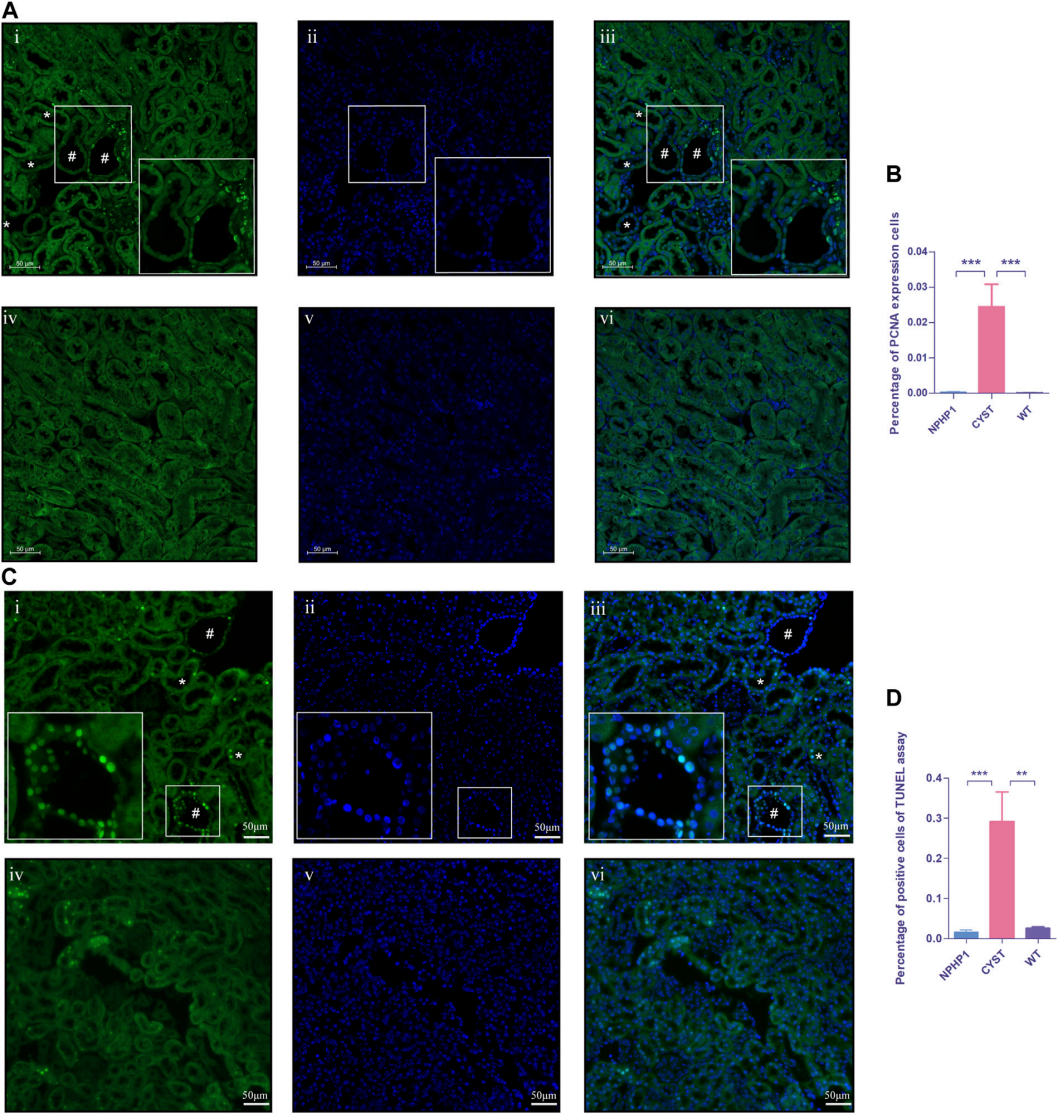
Proliferation and apoptosis of renal cyst cells and tubular epithelial cells of NPHP1 and WT mice. **(A)** PCNA expression in the cyst cell nuclei was increased in NPHP1 mouse renal cyst cells compared with uninvolved tubular cells of NPHP1 mice and normal tubular cells in WT mice. Nuclei were stained with DAPI (blue) and PCNA (green). (i–iii: NPHP1 mice; iv–vi: WT mice; immunofluorescence, scale bar: 50 μm); # point to renal cyst, * point to dilated tubule. **(B)** The percentage of PCNA-expressing cells was quantified. (*p* < 0.001). **(C)** Detection of apoptosis of renal tubular epithelial cells and cystic cells. Sections were stained with DAPI (blue) and FITC (green). Green fluorescence for nuclear labeling of apoptotic cells. (TUNEL assay; i–iii: NPHP1 mice, iv–vi: WT mice; Scale bar: 50 μm); # point to renal cyst, * point to dilated tubule. **(D)** The percentage of TUNEL-positive cells was quantified by tubular cells of CYST, NPHP1 and WT, respectively. (*p* < 0.001) (CYST, cyst cells; NPHP1, renal tubular cells in NPHP1 mouse specimens, excepting cyst cells; WT, all renal tubular cells in wild-type mouse specimens. *n* = 6 of each group).

An imbalance in the regulation of proliferation and apoptosis in renal cyst cells has been recognized as an important factor contributing to cyst formation in ADPKD and NPH(Sugiyama and Yokoyama, 2006; Ibrahim, 2007; Lee, 2016; Shigeta et al., 2018). Our snRNA-seq results showed that *PCNA*, *Cyclin D1* and *Caspase3* levels were increased in cyst cells compared to all other kidney cells (Supplementary Figure S2). Immunofluorescence assays revealed that the PCNA expression in the nuclei of renal cyst cells and dilated tubule cells of NPHP1 mice was slightly increased compared with that in other tubular cells in NPHP1 mice and normal tubular cells in WT mice (Figures 6A, B). TUNEL assays revealed that a proportion of cyst cells and dilated tubule cells had significantly increased apoptotic nuclei, whereas the remaining tubule cells of NPHP1 mice and all normal tubule cells of WT mice had only a small number of apoptotic nuclei (Figures 6C, D). These results indicated that enhanced proliferation and apoptosis in NPHP1 kidney cyst cells may contribute to the formation of cysts.

### 3.7 Interrogation of cell‒cell interactions between cyst cells and non-cyst cells

**FIGURE 7:**
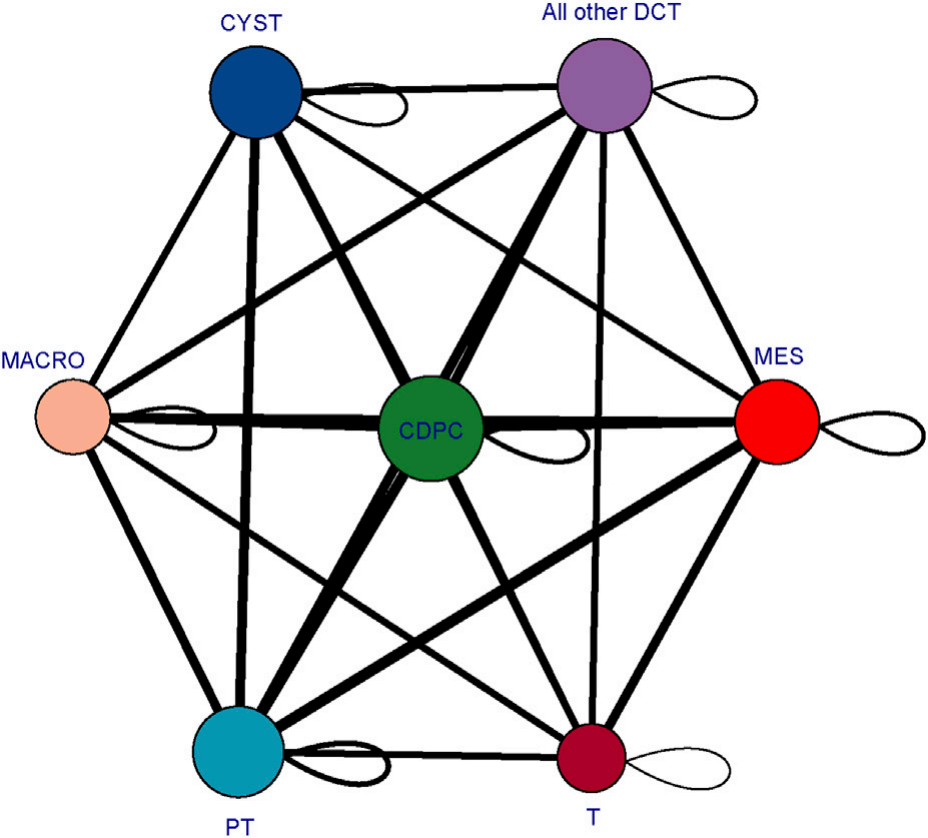
Cellular interaction network diagram. The larger the bubble is and the greater the total number of bubbles is, the stronger the association of the subpopulation in the population; the thicker the line is, the greater the number of significantly enriched ligand‒receptor pairs between subpopulations and the stronger the communication relationship between subpopulations. (CYST, cyst cells; all other DCT, all other distal convoluted tubule cells except cyst cells; PT, proximal tubule cells; MES, mesangial cells; CD-PC, collecting duct principal cells; MACRO, macrophages; T, T lymphocytes).

To determine the mechanisms driving cyst cell formation, we performed cell‒cell interaction analyses for several major cell types. In total, we identified 409 pairs of interactions. We found that all the cell subpopulations were interrelated (Figure 7). We focused on exploring the relationships between cyst cells and the other cell types, and we found that CDPC, PT, and the rest of the DCTs express Fgf9, which can bind to the Fgfr2 receptor in cyst cells; FGF9/FGFR2 interactions have been reported to induce cell proliferation (Chang et al., 2018). In addition, we found that the SPP1/CD44 interaction pair stood out among the immune–cyst cell interaction pairs; i.e., macrophages and T lymphocytes specifically highly express CD44, which interacts with osteopontin (OPN) encoded by the SPP1 gene expressed by cyst cells. The SPP1/CD44 interaction has been shown to be associated with promoting cell proliferation and stemness of tumor cells (Wang et al., 2020; Nallasamy et al., 2021). These findings suggest that tubular epithelial cell-mediated FGF9/FGFR2 and immune cell-mediated SPP1/CD44 interactions may promote cyst formation.

## 4 Discussion

Cyst formation in the kidney is a common feature of renal ciliopathies. Most studies on renal cystogenesis have focused on ADPKD, the major hereditary human kidney disease. In contrast, research on cystogenesis in NPH is rare, probably due to the lack of a suitable NPHP1 animal model. Typically, renal cyst cells tend to have a flattened or cuboidal shape and sparse cytoplasm (Chen et al., 2020). Studies of ADPKD kidneys had shown that cells in individual cysts may arise by clonal proliferation from a single precursor, resembling traits of benign neoplasms (Pei, 2001; Bowden et al., 2021). Both increased proliferation and apoptosis have been reported as features of cystic cells in both ADPKD and NPH(Tao et al., 2005; Wodarczyk et al., 2010; Fan et al., 2013; Lee, 2016; Mannella et al., 2019).

Cystic cells may arise either from the dedifferentiation of mature tubular cells or from progenitor cells. Indeed, they continued to express markers of early development and dedifferentiation, such as *Pax2*, in both ADPKD and NPHP1 patients (Murer et al., 2002; Stayner et al., 2006; Grimley and Dressler, 2018), as also found in NPHP1 mice (Supplementary Figures S3A, B). The analysis of single-cell subpopulations is a powerful way to distinguish between these two possibilities.

By generating a high-resolution cell atlas of the NPHP1 kidney using snRNA-seq and conducting an unbiased analysis of single-cell transcriptomes, we found that virtually all cell types had detectable transcriptional responses to the deletion of NPHP1. Most notably, there was a significant increase in the DCT3 subpopulation, which showed specific downregulation of genes regulating tubular development and morphogenesis, such as *Calb1*, *Npnt* and *Shank2*. Analysis of the differentiation trajectories of individual cells further revealed that the cystic cells retained at an earlier stage of development. This could suggest that these cells were ‘arrested’ at an immature stage and failed to develop into mature distal tubule cells. Cells at earlier developmental stage have talent of higher proliferative activity and may clonally proliferate to form renal cysts. Our findings of a distal origin were consistent with previous microdissection studies showing that NPH cysts primarily arised from distal convoluted and medullary collecting tubules(Sherman et al., 1971).

We next identified *Niban1*, a gene preferentially expressed in cystic cells, as a specific marker of cystic cells. *Niban1* is highly expressed in some cancer cells and may serve as a prognostic marker for certain cancers, such as renal and thyroid carcinoma (Majima et al., 2000; Adachi et al., 2004; Nozima et al., 2019). Northern blot analysis of human tissues showed strong Niban1 expression in the heart, skeletal muscle, pancreas, white blood cells, and prostate, with moderate expression in the colon and spleen. However, no Niban1 expression was detected in healthy human or wildtype rat/mouse kidneys (Majima et al., 2000). *Niban1* is mainly expressed in the cytoplasm and is used as a candidate marker for the development of renal tumors, especially early-stage renal carcinoma (Adachi et al., 2004). *Niban1* promotes tumor cell proliferation by upregulating the expression of *MMP2* and *Cyclin D1*(Zhang et al., 2019) and inhibits apoptosis by regulating *MDM2* and *p53* interactions (Ji et al., 2012). Targeting *Niban1* has tumor suppressive effects (Ayesha et al., 2022). In this study, we found that *Niban1* was specifically expressed in NPHP1 cystic cells, which were characterized by enhanced proliferative and apoptotic activity. *PCNA*, *Cyclin D1* and *Caspase3* were upregulated in cyst cells compared to all other kidney cells. Whether *Niban1* regulates cyst cell formation through the same pathways as in cancer cells remains to be verified. Previous studies had shown that the protein encoded by *NPHP1* had an anti-apoptotic function, that silencing *NPHP1* increases susceptibility to apoptosis, leading to significantly higher levels of apoptosis (Wodarczyk et al., 2010; Shigeta et al., 2018; Mannella et al., 2019). These observations were consistent with the findings of the present study, in which we found that, unlike in ADPKD, apoptosis appears to be more predominant than cell proliferation in NPHP1. Whether this is responsible for the significant difference in cyst and kidney size between NPH and ADPKD requires further investigation by subsequent quantitative and correlative analysis.

Furthermore, we found that the interaction of FGFR2/FGF9 in tubular cells and cyst cells may play an important role in the induction of cyst cell formation. FGFRs can be activated by FGFs to induce cell proliferation in normal chondrocytes, pancreatic cancer cells, non-small cell lung cancer cells and nasopharyngeal carcinoma cells (Matsuda et al., 2014; Bradley et al., 2015; He et al., 2016; Hu et al., 2016). In mouse Leydig tumor cells, FGF9 induces the expression of FGFR1-4 and interacts with FGFR2 to promote cell proliferation by activating the ERK1/2 pathway (Chang et al., 2018). In addition to FGFR2/FGF9, we also found that SPP1/CD44 interactions between immune cells, especially macrophages, and cyst cells may play an important role in the induction of cyst cell formation. Studies had shown that SPP1/CD44 signaling in the perivascular niche promotes the stem cell-like properties of glioma cells and that increased SPP1 expression induces GBM-associated macrophage infiltration (Pietras et al., 2014; Wei et al., 2019). In the present study, cyst cells showed enhanced proliferative activity compared to the other tubule cell types and retained at an earlier developmental stage. Whether this effect is also induced through the FGFR2/FGF9 and SPP1/CD44 interactions deserves further investigation.

Tubulointerstitial inflammation with mononuclear immune cell infiltration and diffuse interstitial fibrosis is another pathological characteristic of NPH and usually emerges during the early stages of disease (Wu et al., 2016; Docherty et al., 2019; Jin et al., 2020). In NPH, a recent study (Quatredeniers et al., 2022) showed a significant infiltration of immune cells in the kidneys of NPHP1 patients, with macrophages (CD68-positive cells) being the predominant immune cells, as well as neutrophils and T cells. In ADPKD, after knockdown of *Pkd1* or *Pkd2,* C-C motif chemokine ligand 2 (CCL2) was highly upregulated at the onset of cyst formation (Karihaloo et al., 2011). Cassini MF et al. (Cassini et al., 2018) demonstrated that macrophage aggregation promoted ADPKD cyst growth, a process that was regulated by CCL2 and can be alleviated by CCL2 or CCR2 inhibitors. Elevated levels of CCL2 were also observed in tubular cells of NPHP1 patients. A murine model of *Lkb1* inactivation in renal tubules developed an NPHP1-like phenotype in humans and showed a high level of CCL2 in renal tubular cells and macrophage recruitment. However, the renal phenotype and immune cell recruitment are independent of CCL2 upregulation(Viau et al., 2018; Quatredeniers et al., 2022). In our NPHP1 mouse model, the macrophage population was increased significantly compared with that in WT mice. GO analysis of macrophages showed an association with mitochondrial ATP synthesis as well as energy transport, which indicates that macrophages were activated after *NPHP1* knockdown. A comparison of the marker genes of different macrophage subtypes revealed that the macrophages isolated in our study were M2b-like macrophages (CD68+/CD86+) (Supplementary Figure S4). We also identified increased levels of *CCL2* in NPHP1 kidneys (data not shown). However, the interrogation of cell‒cell interactions between macrophages and cyst cells did not show a relationship between *CCL-2* and cyst cells. Nevertheless, the relationship between renal immune cell infiltration and renal phenotype in NPHP1 needs to be further verified.

In conclusion, our study provides the first comprehensive cell atlas of the NPHP1 kidney coupled with data characterizing the transcriptomic changes in different renal cell subpopulations at the single-cell level. We identified and characterized a subpopulation of renal cyst cell. These findings gave an insight into renal cyst formation in NPHP1. Further researches on the mechanism of renal cysts formation will facilitate new therapy approaches of this disease.

## Data Availability

The datasets presented in this study can be found in online repositories. The names of the repository/repositories and accession number(s) can be found below: NCBI Biosample under SAMN35578313, SAMN35578314, SAMN35578315, SAMN35578316.
