# Supplementary figures and images for: Identification of renal cyst cells of type I Nephronophthisis by single-nucleus RNA sequencing
Source: Front Cell Dev Biol. 2023 Jul 31;11:1192935. doi: 10.3389/fcell.2023.1192935 (PMC10423821; doi:10.3389/fcell.2023.1192935)

# Top 20 of GO Enrichment

GOterm

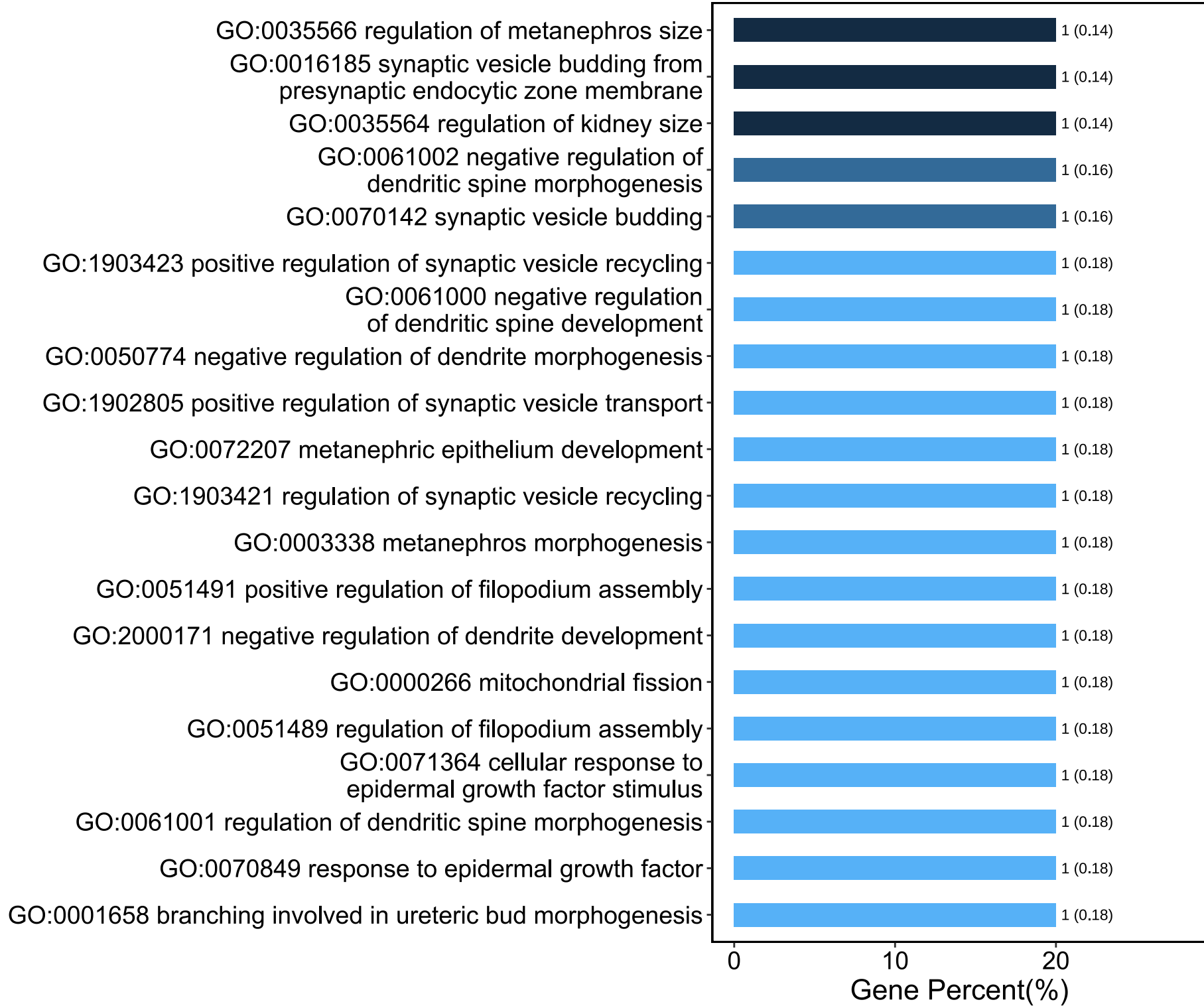

Supplement: Supplementary file 1 [file DataSheet3.ZIP › Gene Ontology (GO) analysis of the upregulated genes in each cluster/Cluster_ATL.P.barplot.pdf]

# Top 20 of GO Enrichment

GOterm

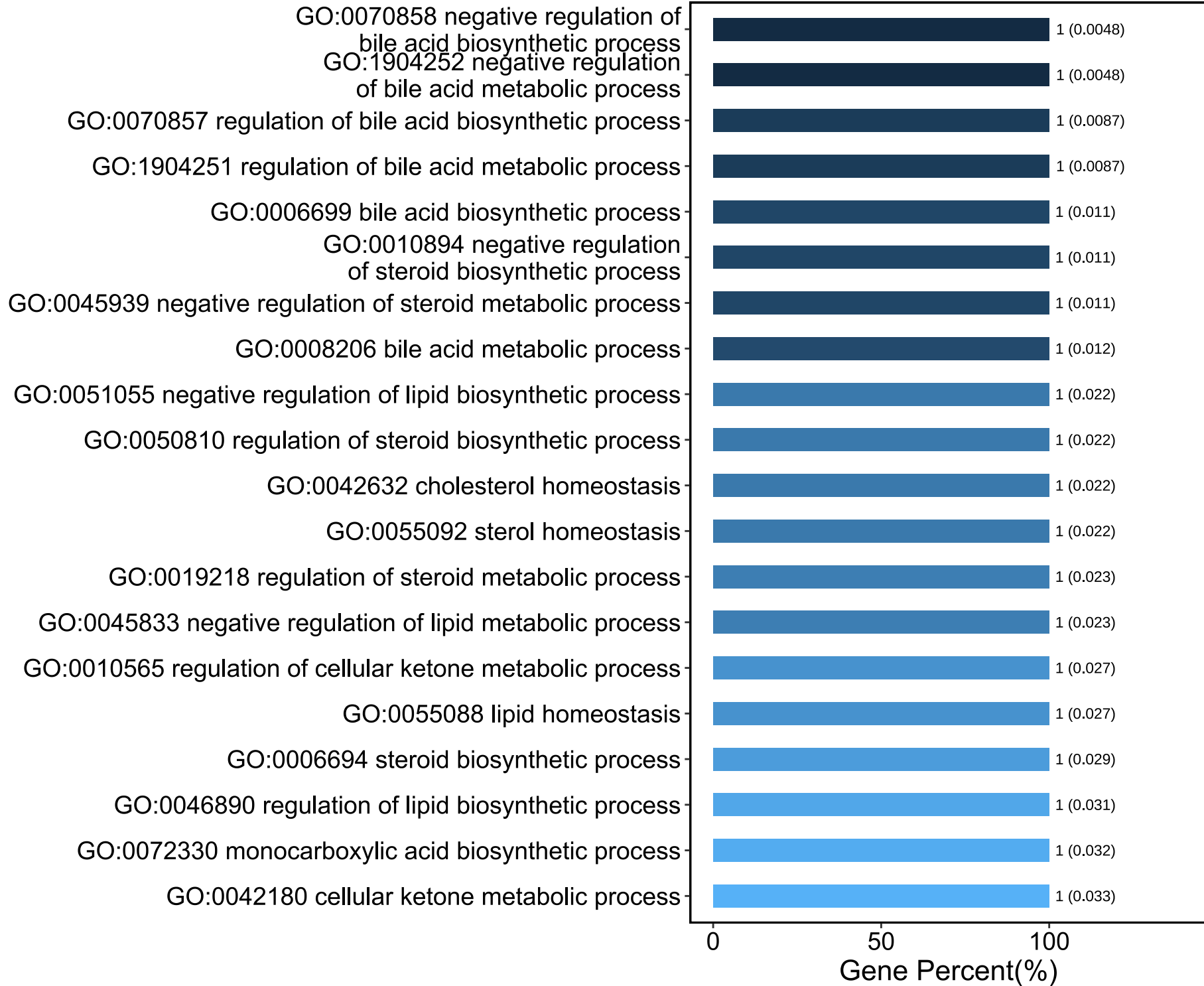

Supplement: Supplementary file 1 [file DataSheet3.ZIP › Gene Ontology (GO) analysis of the upregulated genes in each cluster/Cluster_CDIC-A.P.barplot.pdf]

# Top 20 of GO Enrichment

GOterm

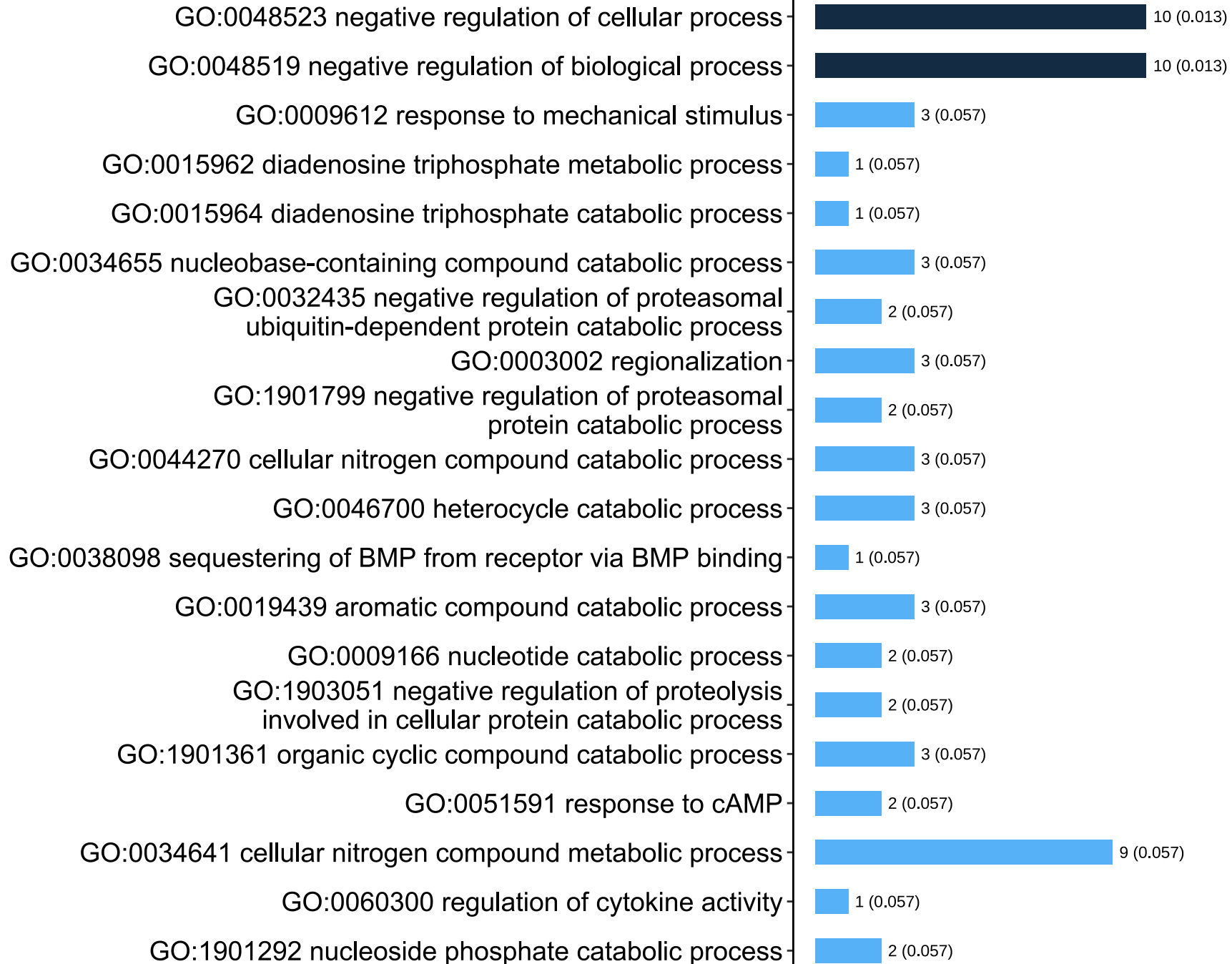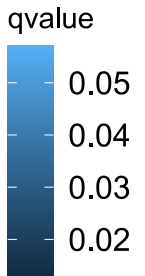

0 30 60 90 120  
Gene Percent(%)

Supplement: Supplementary file 1 [file DataSheet3.ZIP › Gene Ontology (GO) analysis of the upregulated genes in each cluster/Cluster_CDPC.P.barplot.pdf]

# Top 20 of GO Enrichment

GOterm

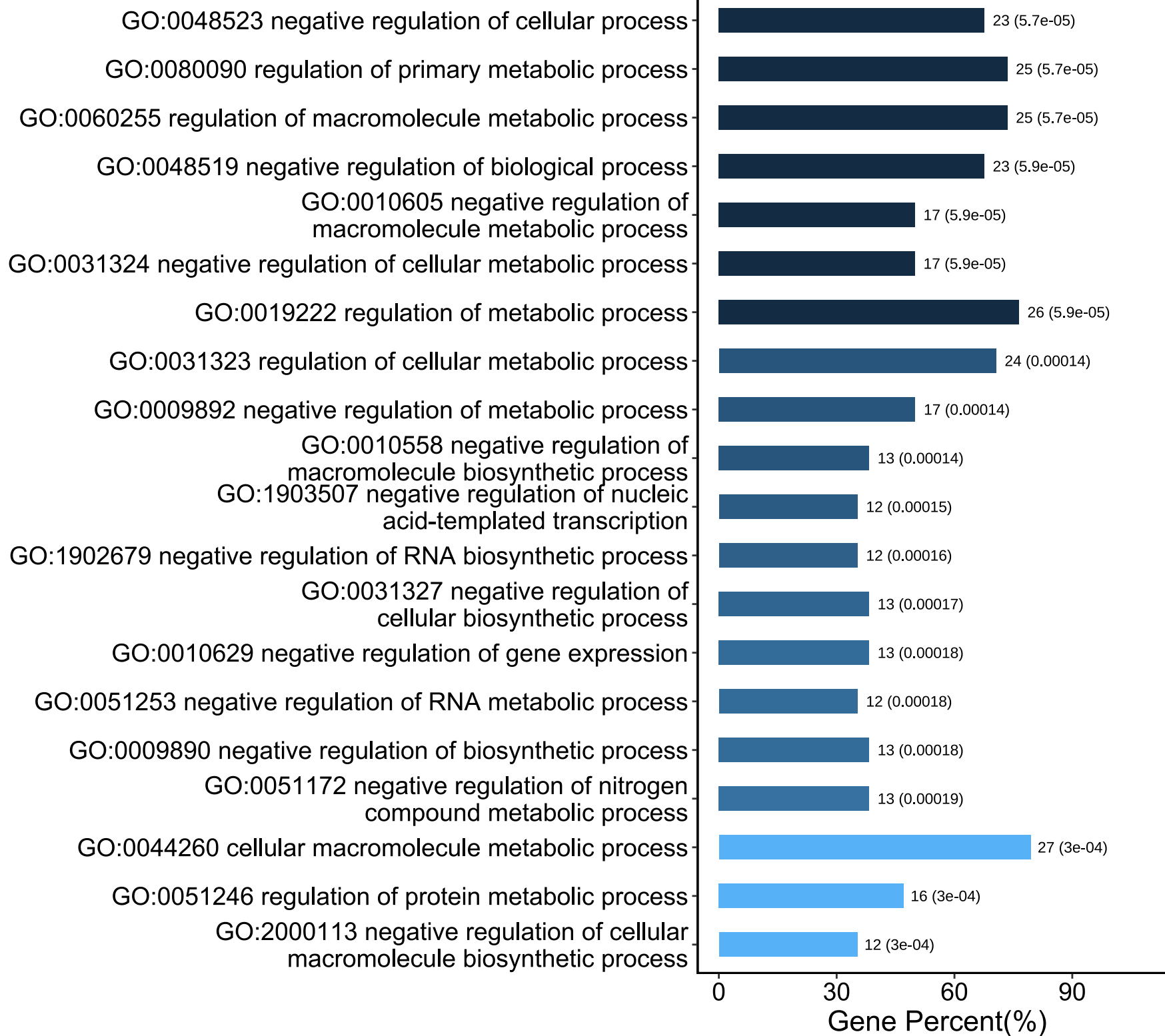

Supplement: Supplementary file 1 [file DataSheet3.ZIP › Gene Ontology (GO) analysis of the upregulated genes in each cluster/Cluster_DCT1.P.barplot.pdf]

# Top 20 of GO Enrichment

GOterm

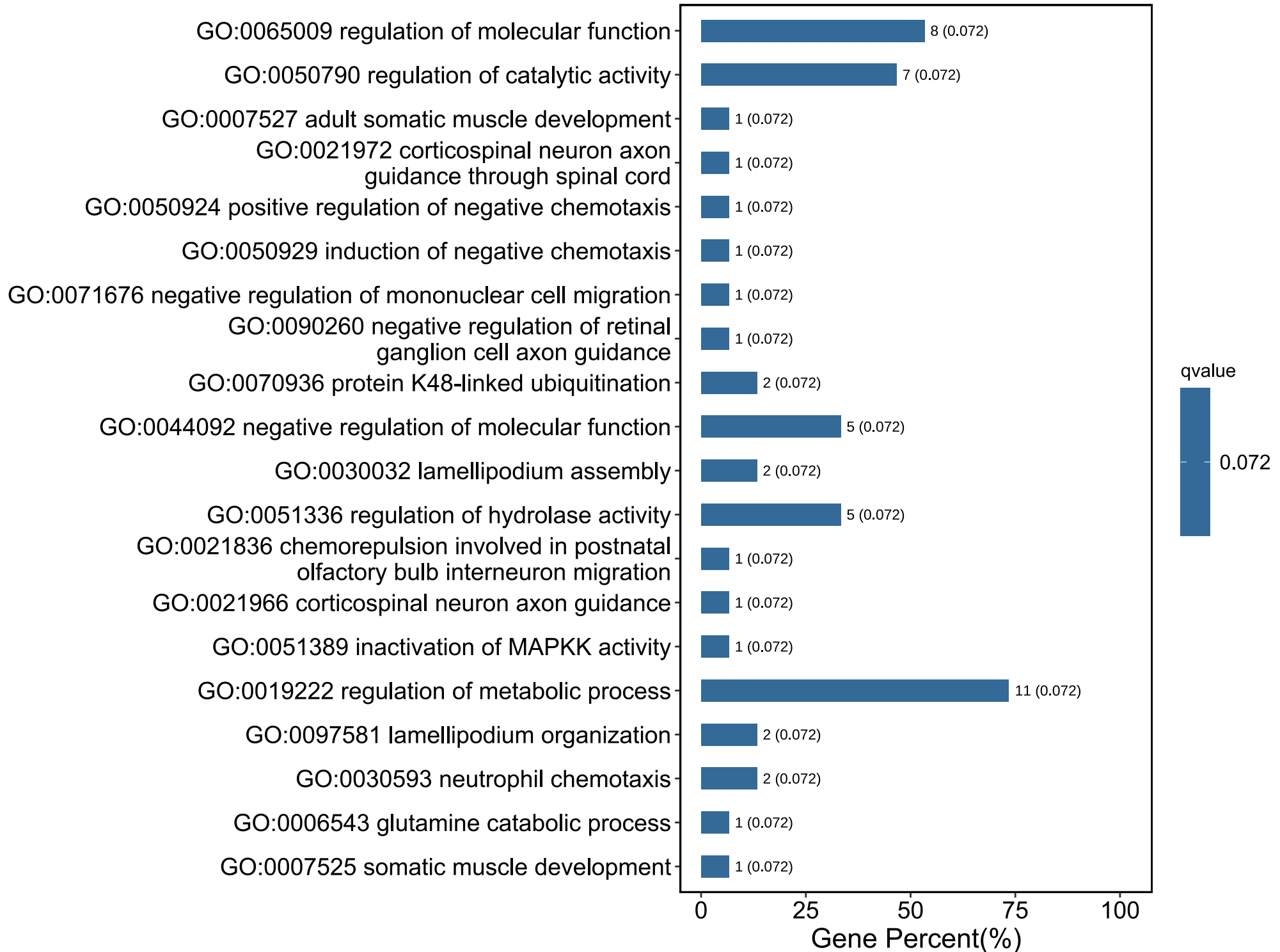

Supplement: Supplementary file 1 [file DataSheet3.ZIP › Gene Ontology (GO) analysis of the upregulated genes in each cluster/Cluster_DCT2.P.barplot.pdf]

# Top 20 of GO Enrichment

GOterm

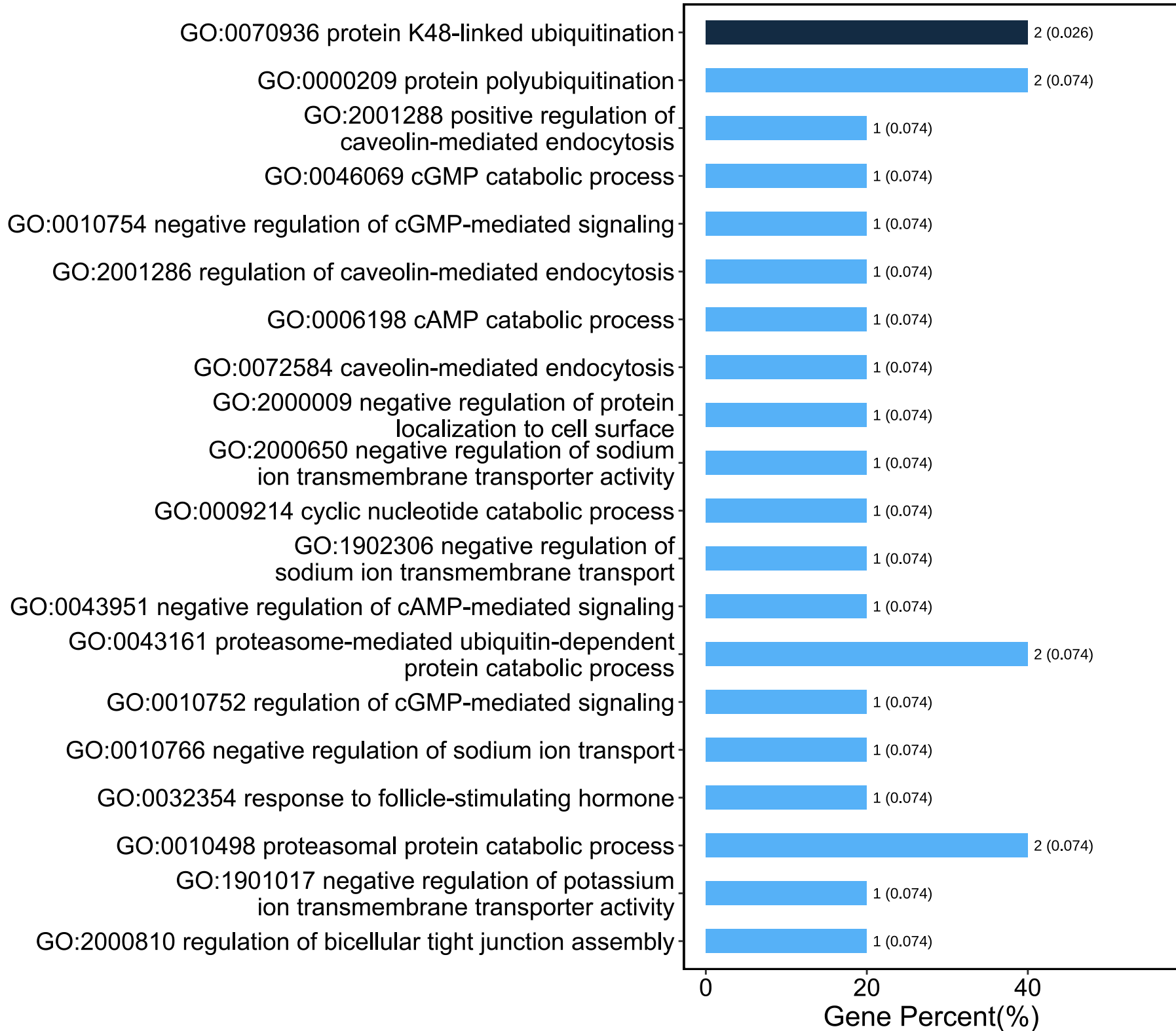

Supplement: Supplementary file 1 [file DataSheet3.ZIP › Gene Ontology (GO) analysis of the upregulated genes in each cluster/Cluster_DCT3.P.barplot.pdf]

# Top 20 of GO Enrichment

GOterm

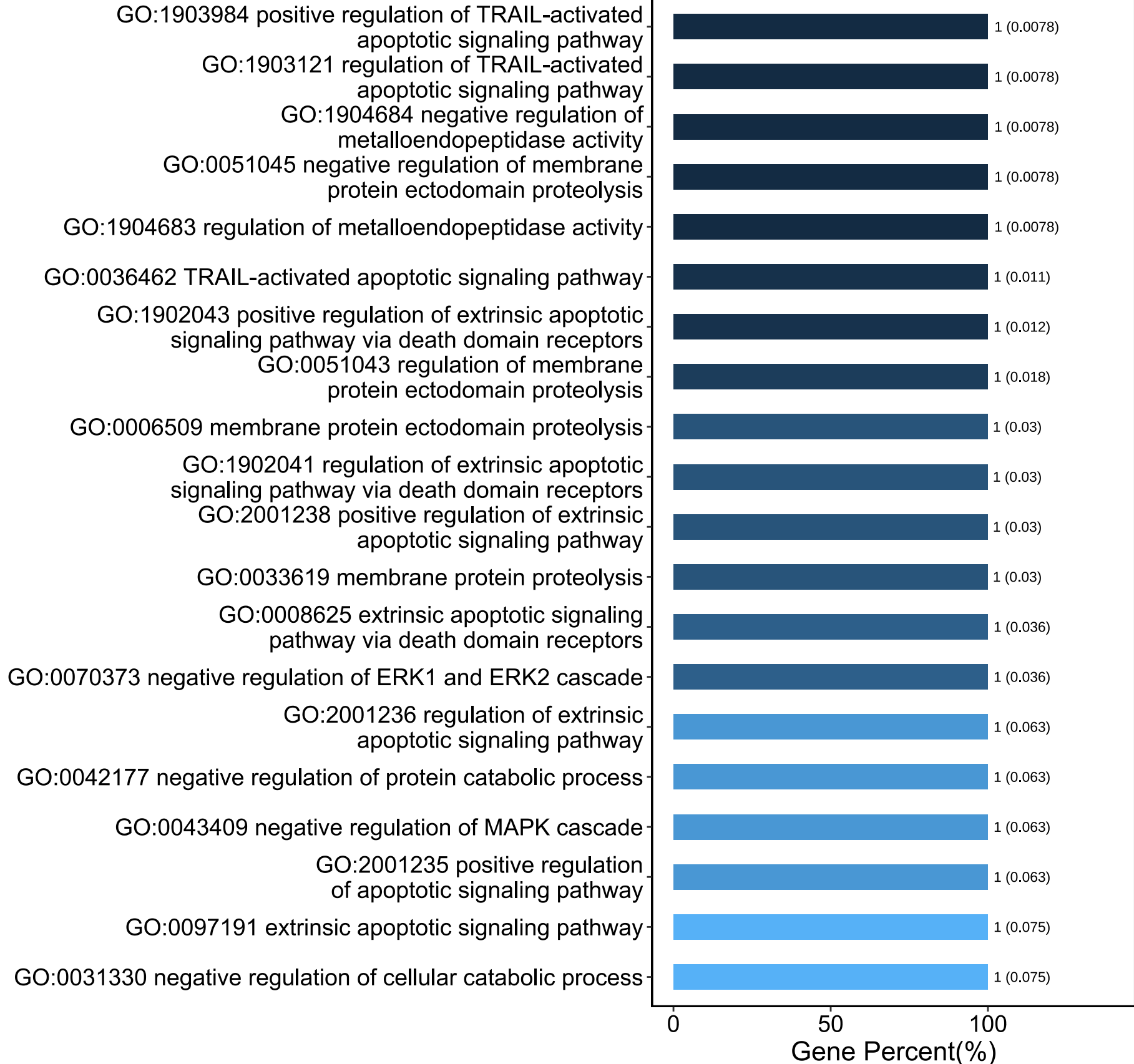

Supplement: Supplementary file 1 [file DataSheet3.ZIP › Gene Ontology (GO) analysis of the upregulated genes in each cluster/Cluster_DTL.P.barplot.pdf]

# Top 20 of GO Enrichment

GOterm

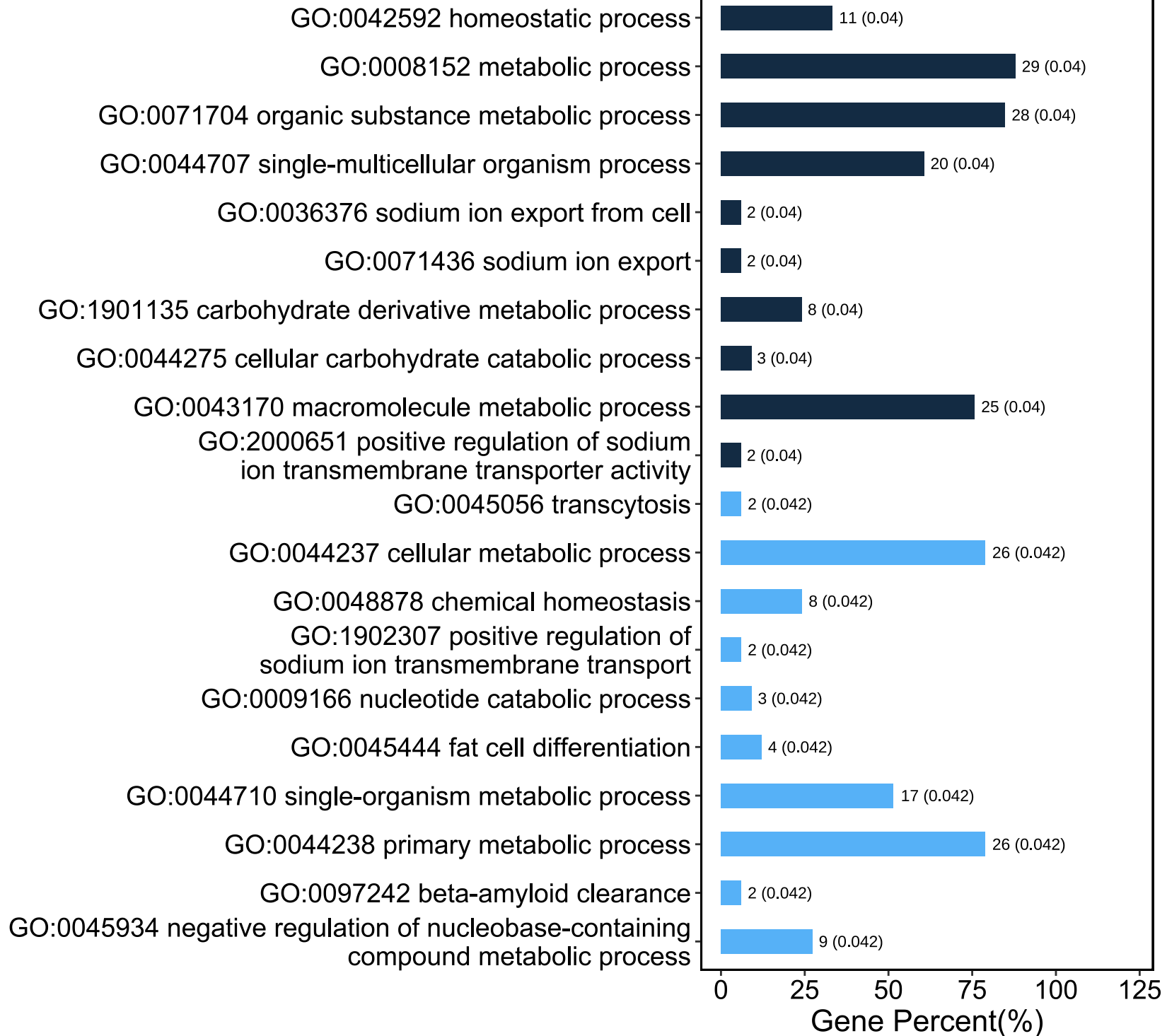

Supplement: Supplementary file 1 [file DataSheet3.ZIP › Gene Ontology (GO) analysis of the upregulated genes in each cluster/Cluster_ENDO.P.barplot.pdf]

# Top 20 of GO Enrichment

GOterm

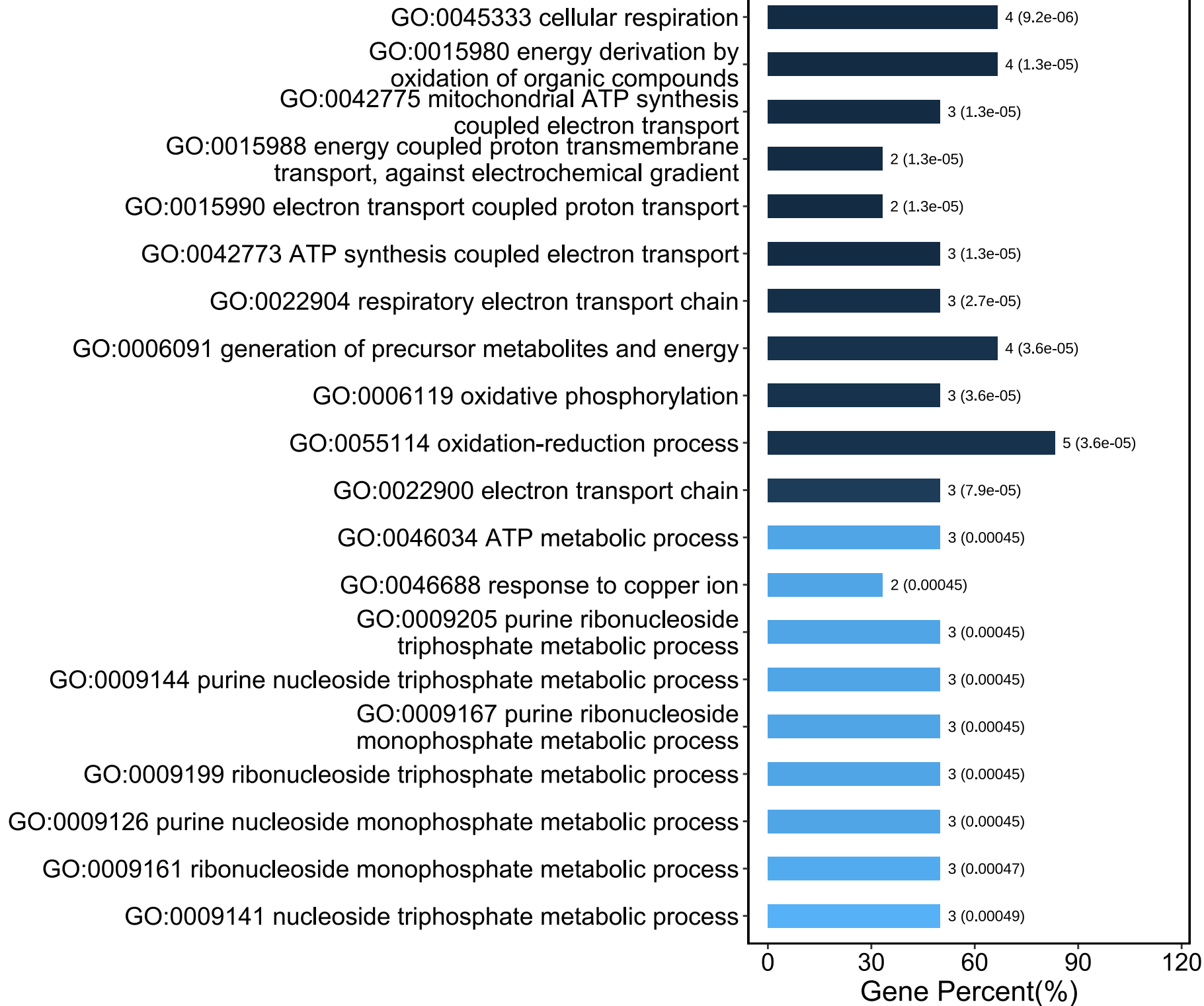

Supplement: Supplementary file 1 [file DataSheet3.ZIP › Gene Ontology (GO) analysis of the upregulated genes in each cluster/Cluster_MACRO.P.barplot.pdf]

# Top 20 of GO Enrichment

GOterm

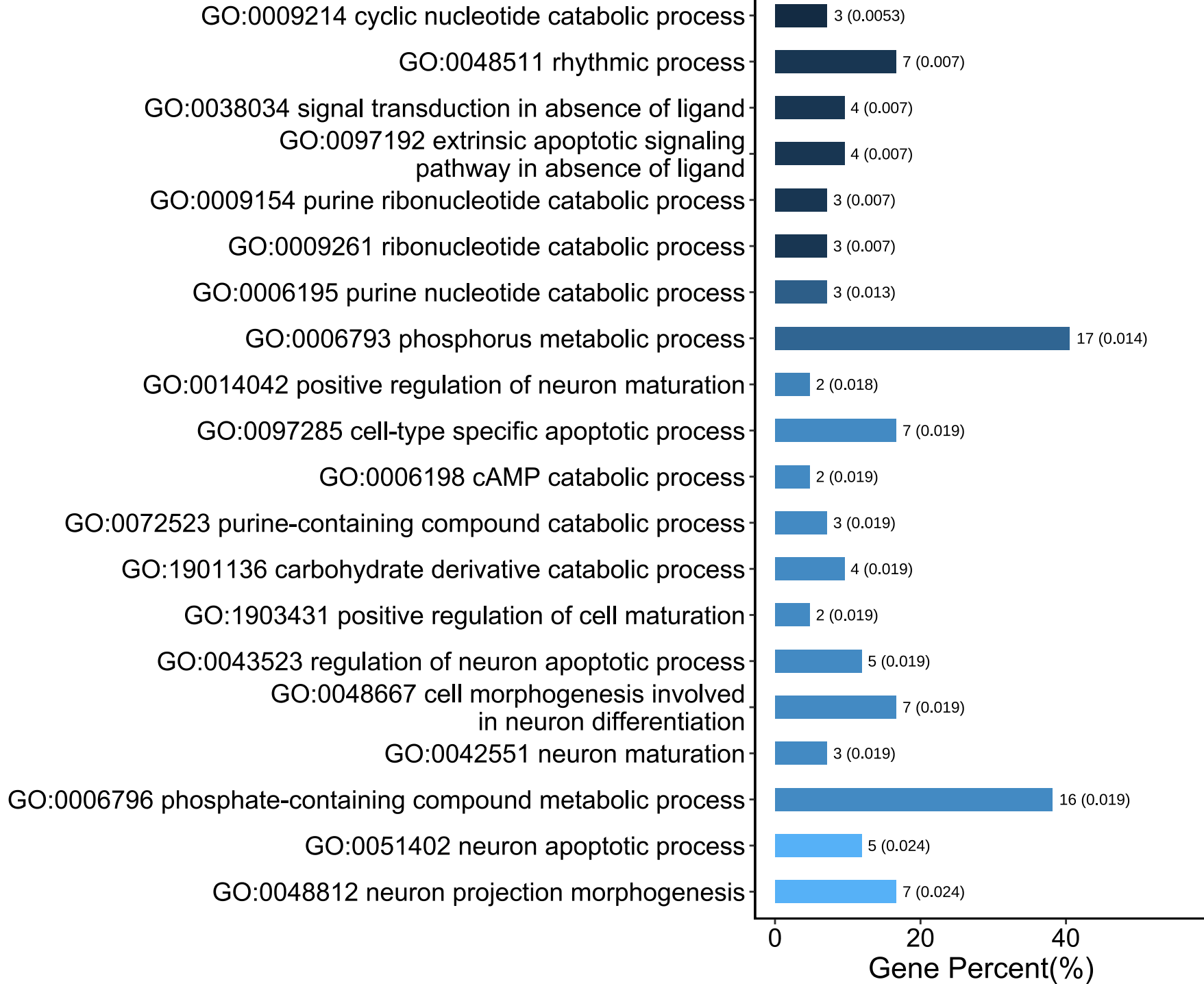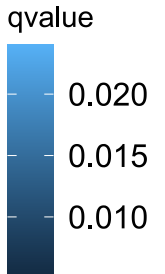

Supplement: Supplementary file 1 [file DataSheet3.ZIP › Gene Ontology (GO) analysis of the upregulated genes in each cluster/Cluster_MES.P.barplot.pdf]

# Top 20 of GO Enrichment

GOterm

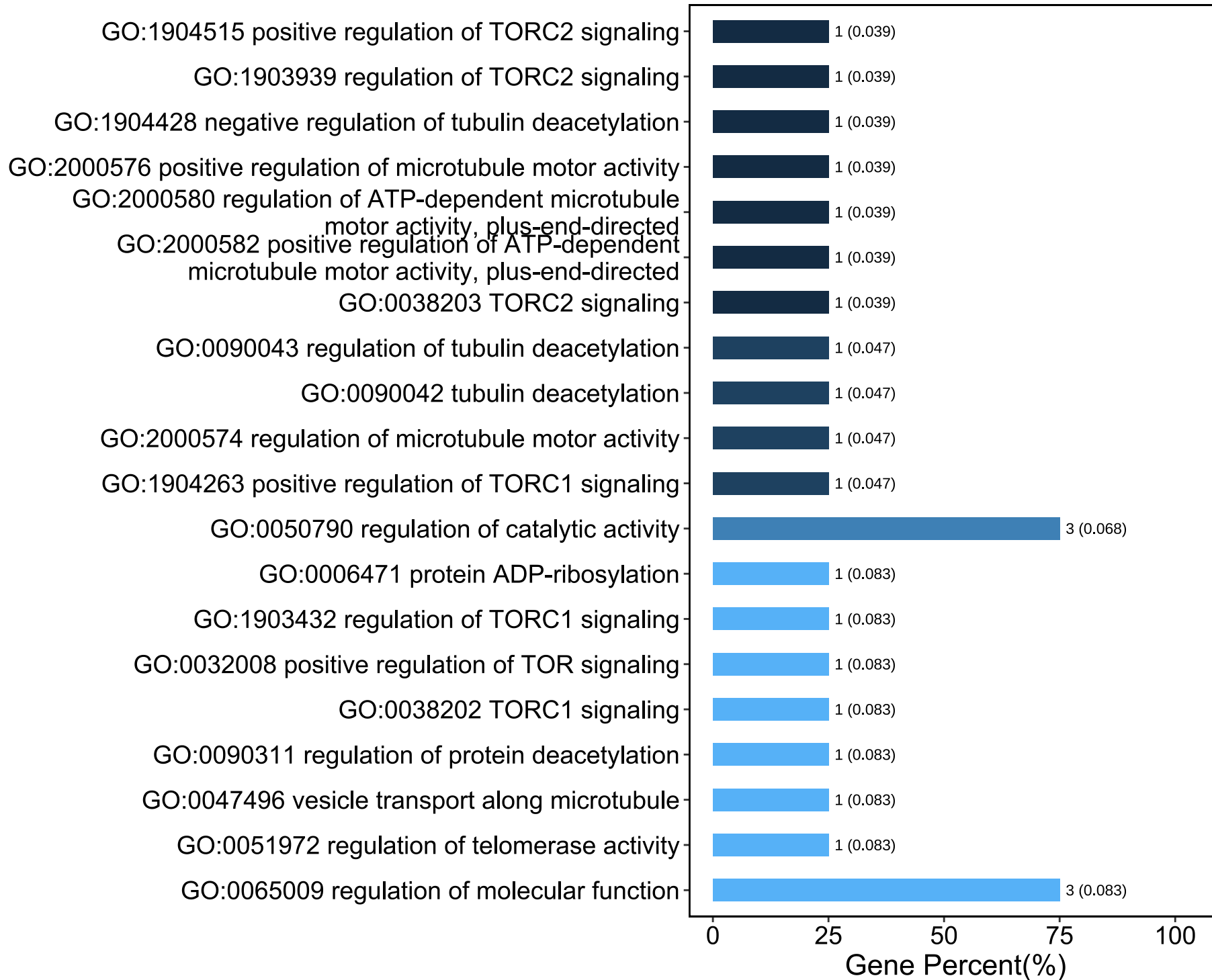

Supplement: Supplementary file 1 [file DataSheet3.ZIP › Gene Ontology (GO) analysis of the upregulated genes in each cluster/Cluster_PODO.P.barplot.pdf]

# Top 20 of GO Enrichment

GOterm

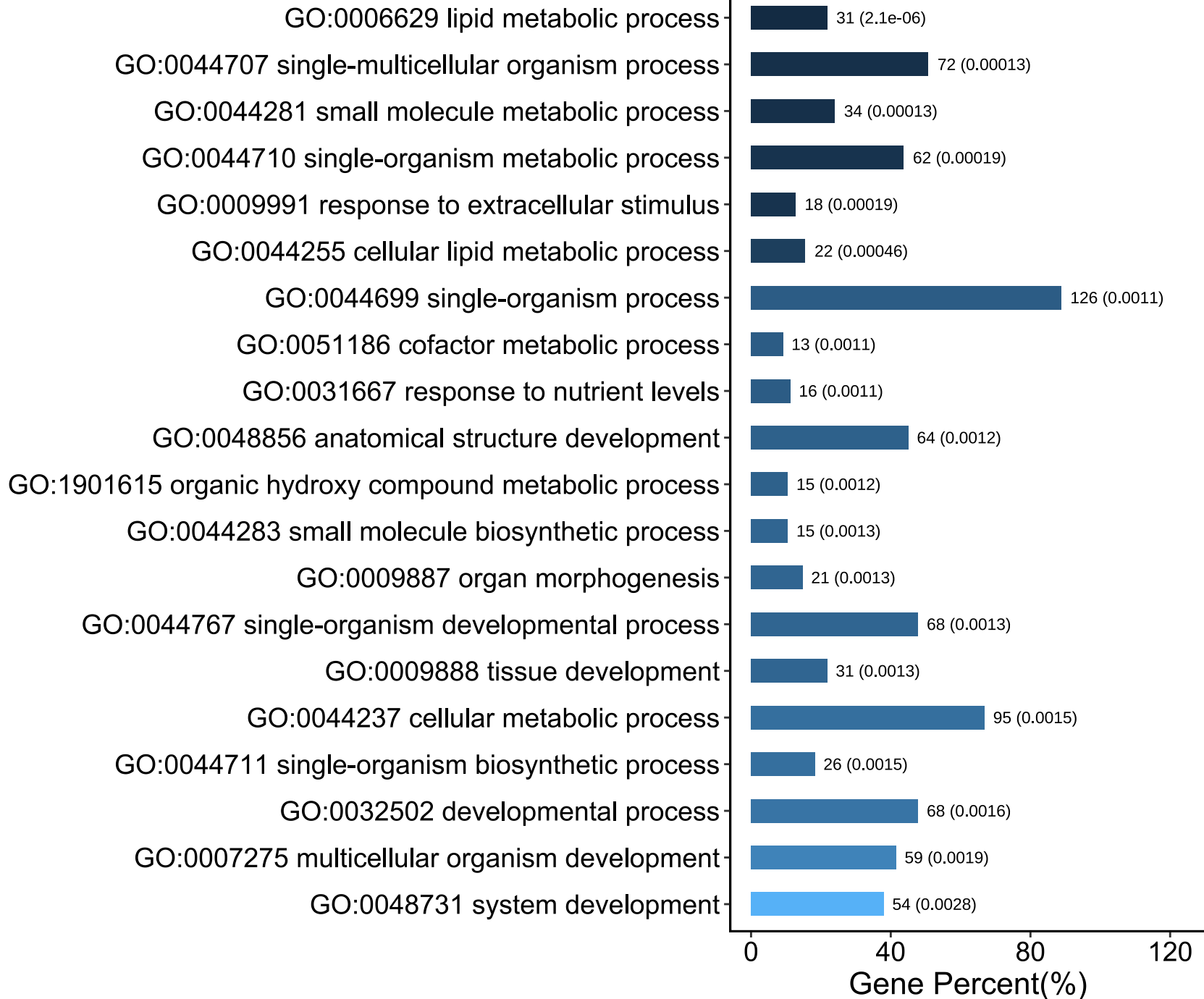

Supplement: Supplementary file 1 [file DataSheet3.ZIP › Gene Ontology (GO) analysis of the upregulated genes in each cluster/Cluster_PT.P.barplot.pdf]

Top 20 of GO Enrichment

GOterm

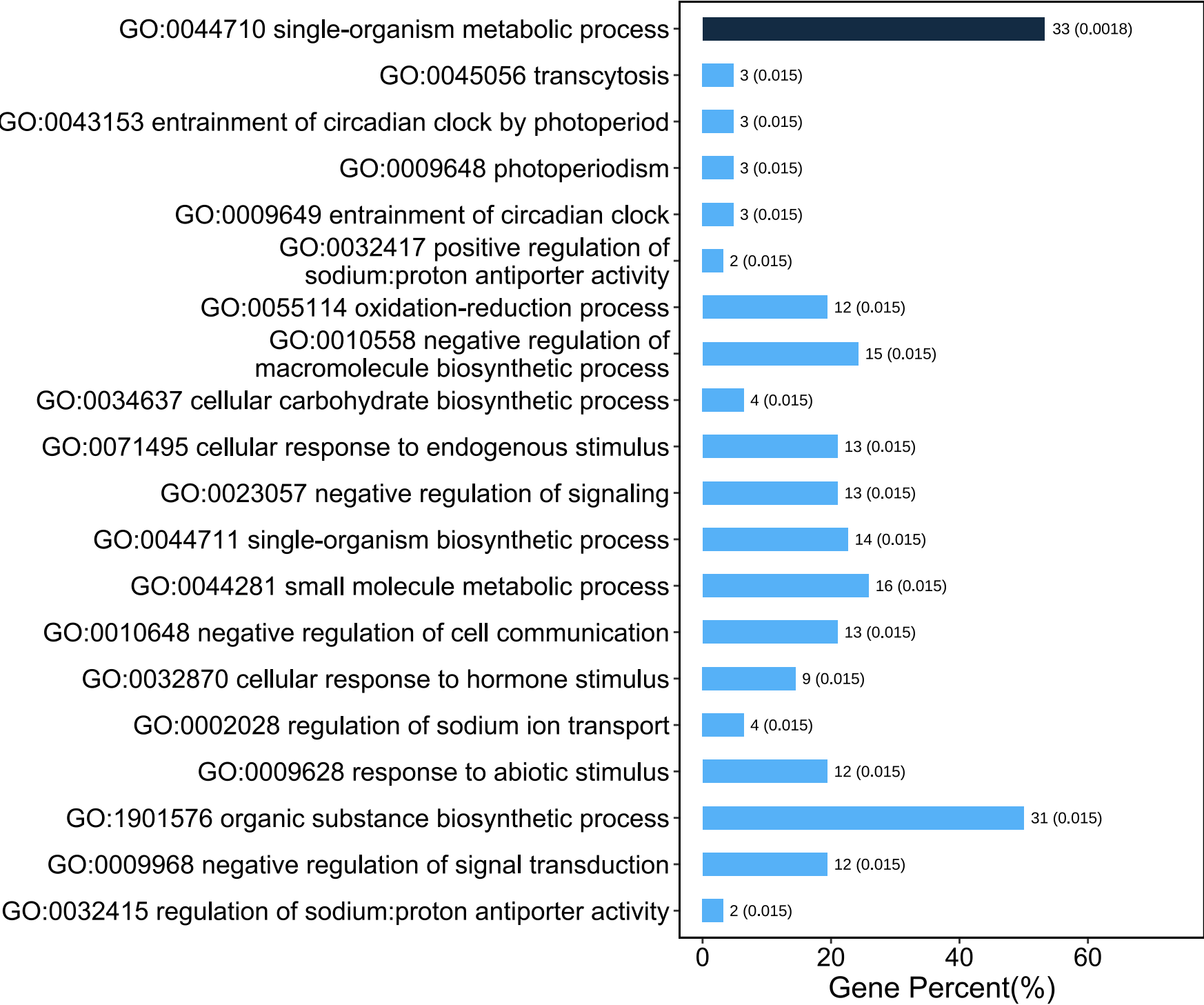

Supplement: Supplementary file 1 [file DataSheet3.ZIP › Gene Ontology (GO) analysis of the upregulated genes in each cluster/Cluster_TAL.P.barplot.pdf]

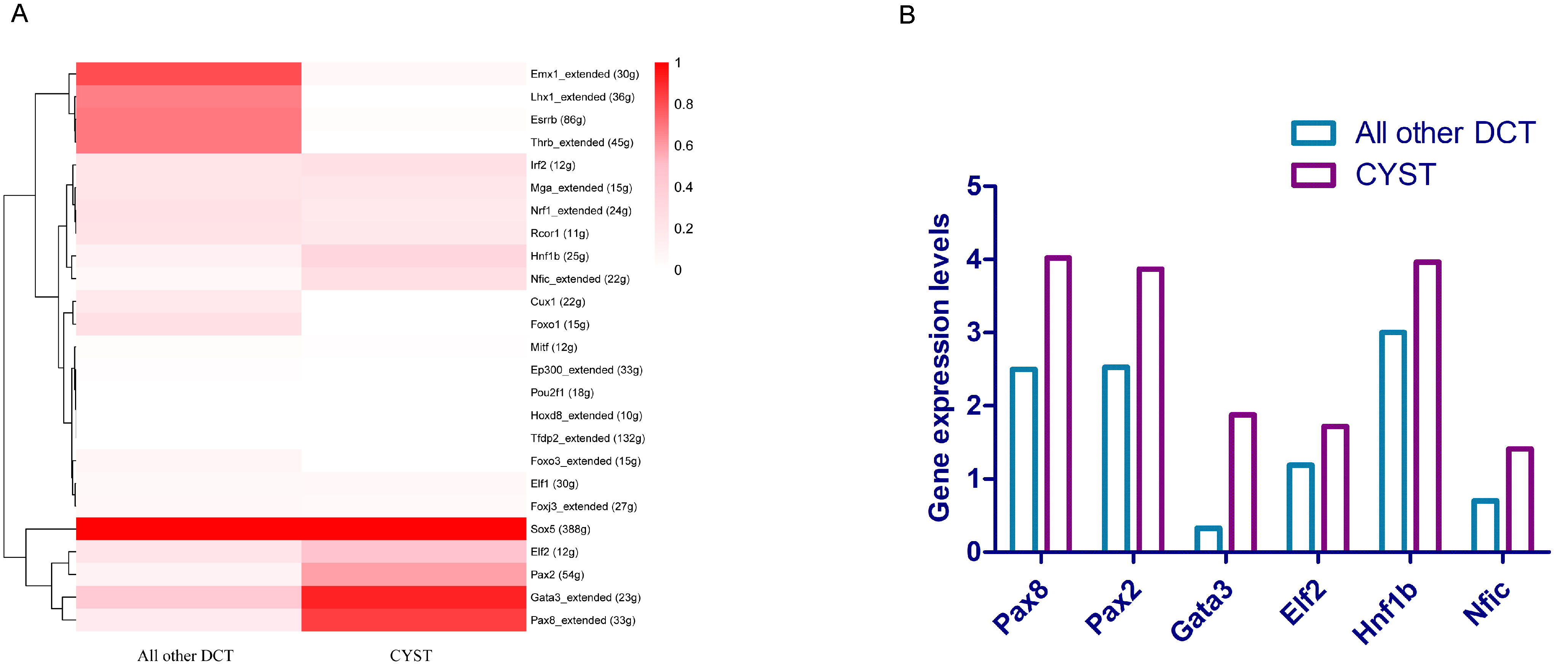

Supplement: Supplementary file 3 [file Image3.TIF]

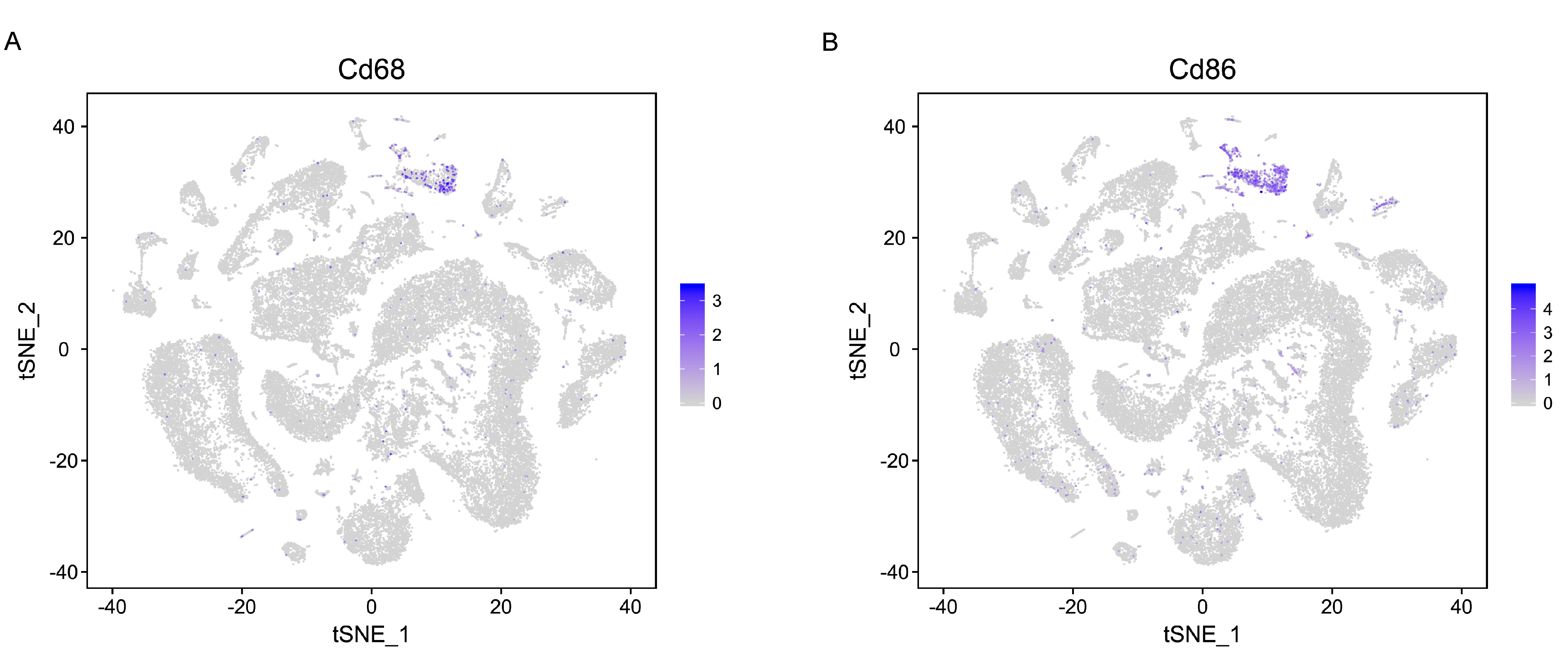

Supplement: Supplementary file 4 [file Image4.TIF]

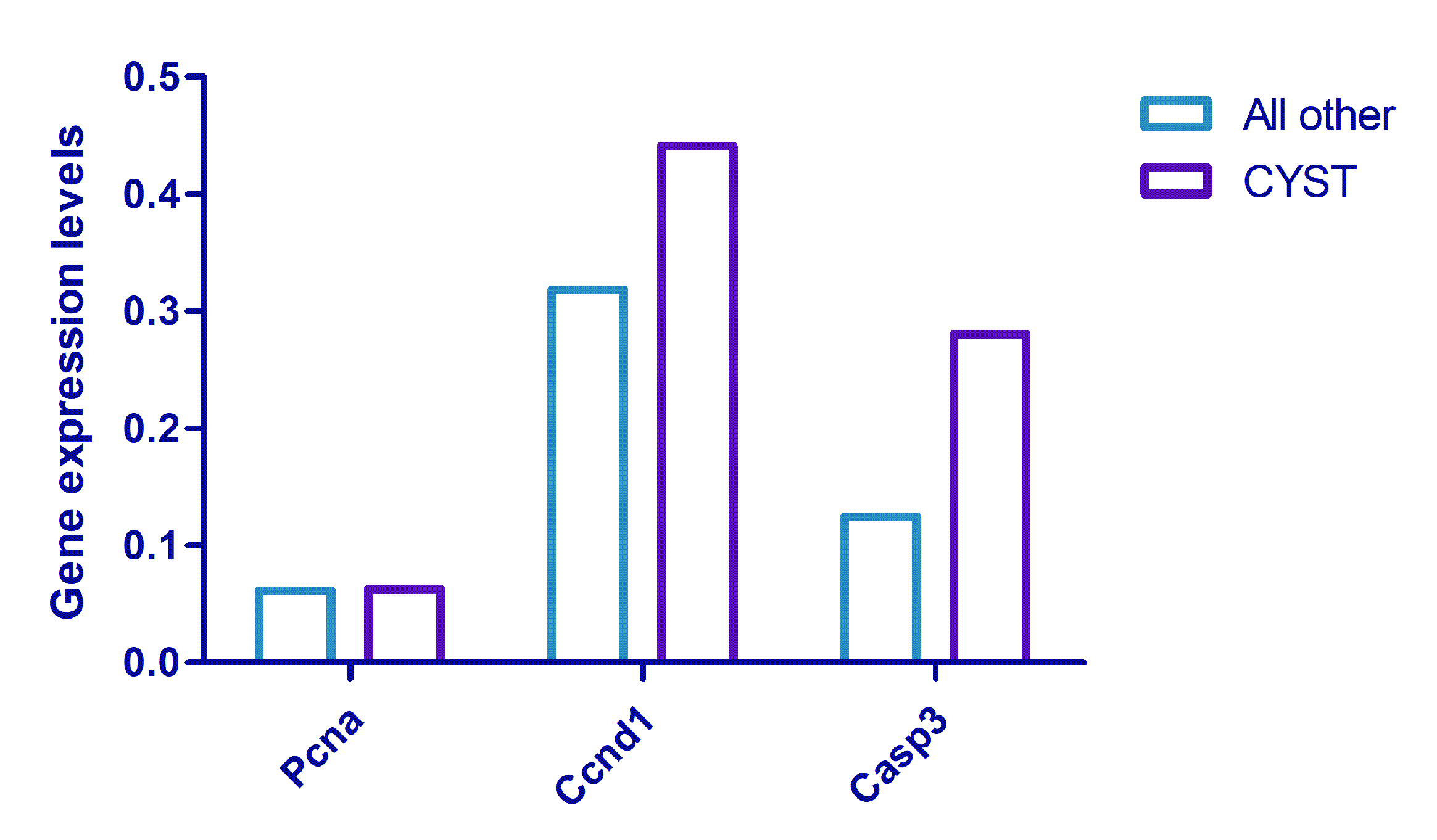

Supplement: Supplementary file 5 [file Image2.TIF]

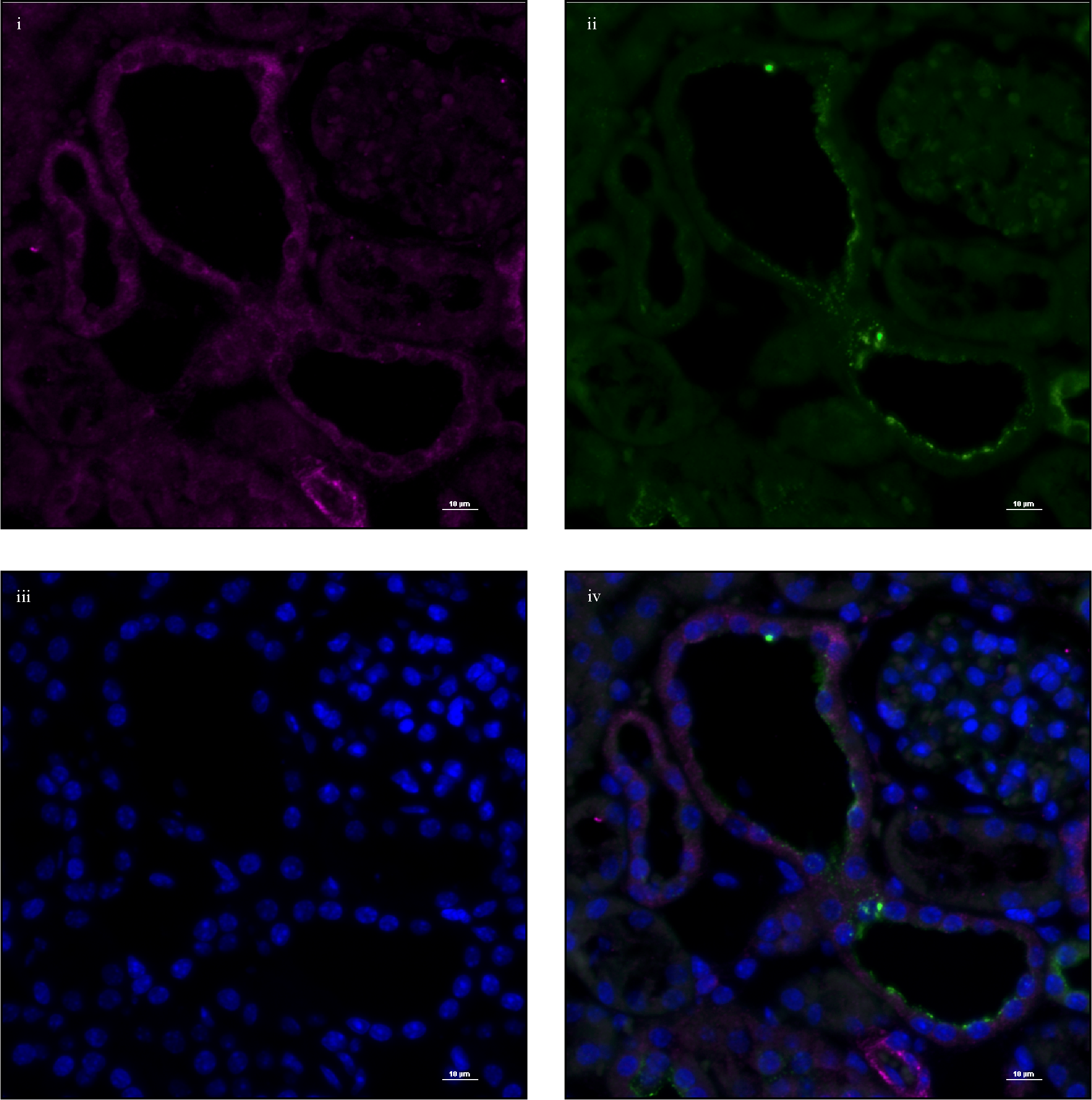

Supplement: Supplementary file 6 [file Image1.TIF]

# Top 20 of GO Enrichment

GOterm

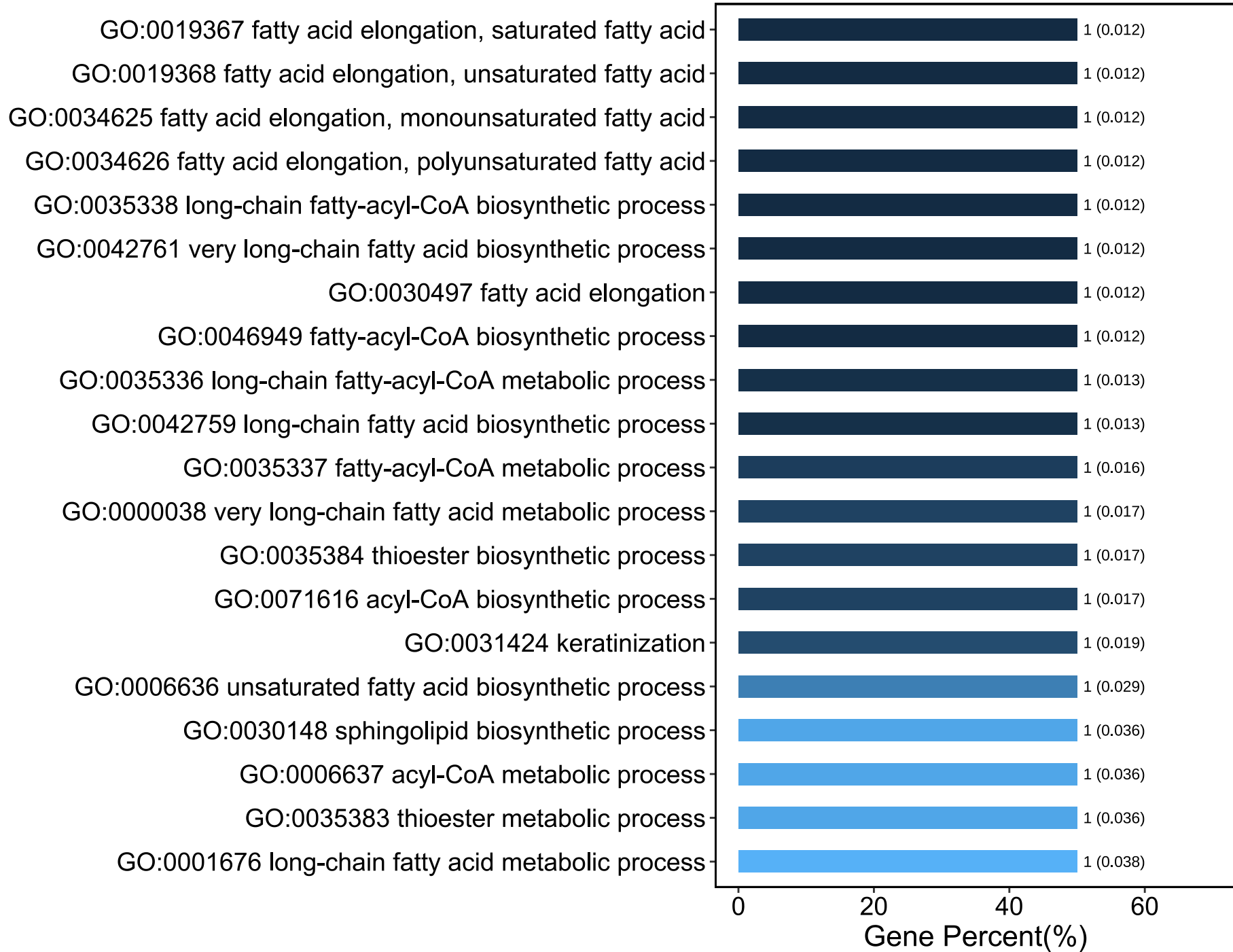

Supplement: Supplementary file 8 [file DataSheet2.ZIP › Gene Ontology (GO) analysis of the downregulated genes in each cluster/Cluster_ATL.P.barplot.pdf]

# Top 20 of GO Enrichment

GOterm

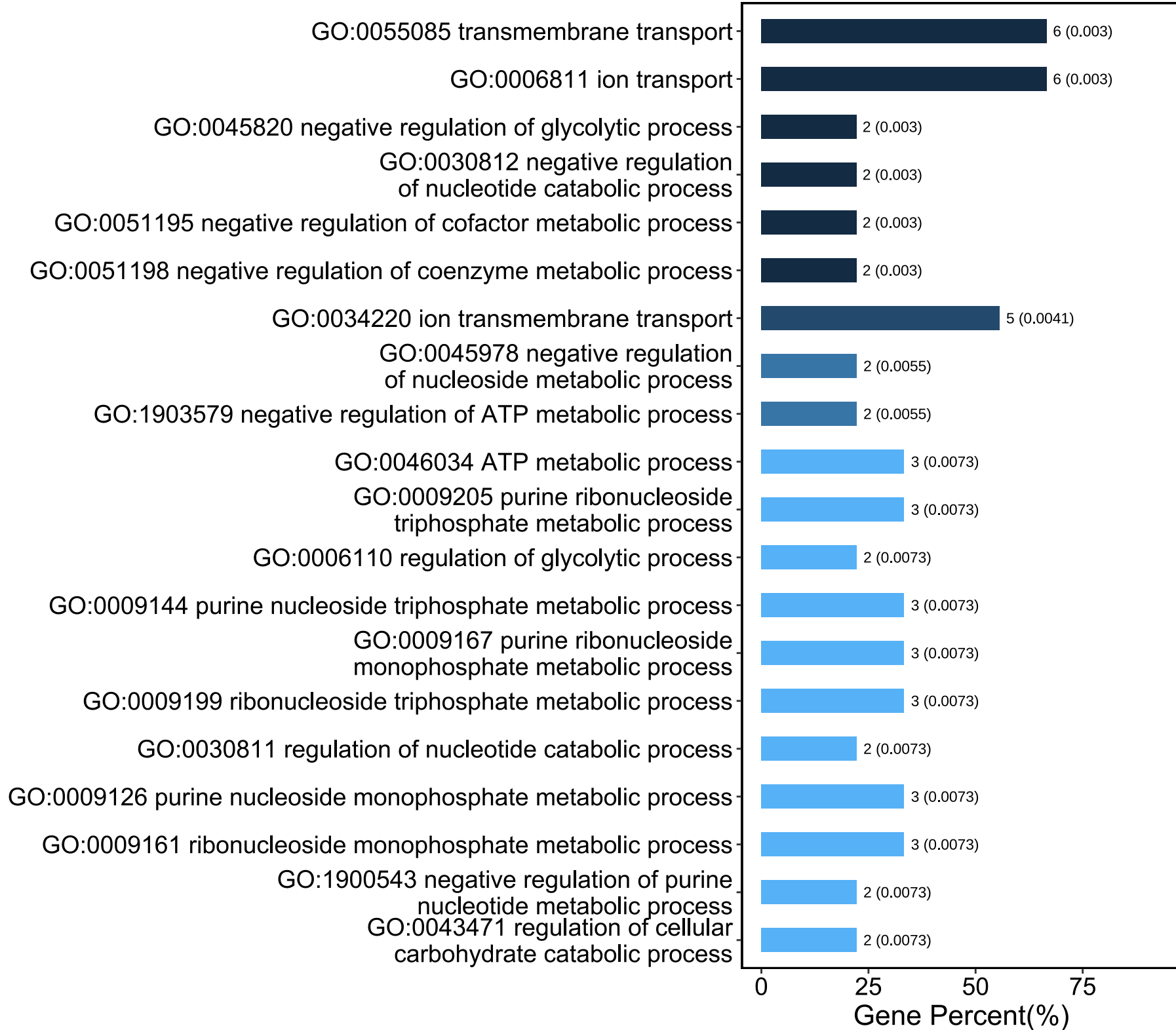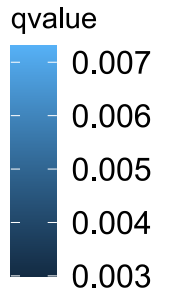

Supplement: Supplementary file 8 [file DataSheet2.ZIP › Gene Ontology (GO) analysis of the downregulated genes in each cluster/Cluster_CDIC-A.P.barplot.pdf]

Top 20 of GO Enrichment

GOterm

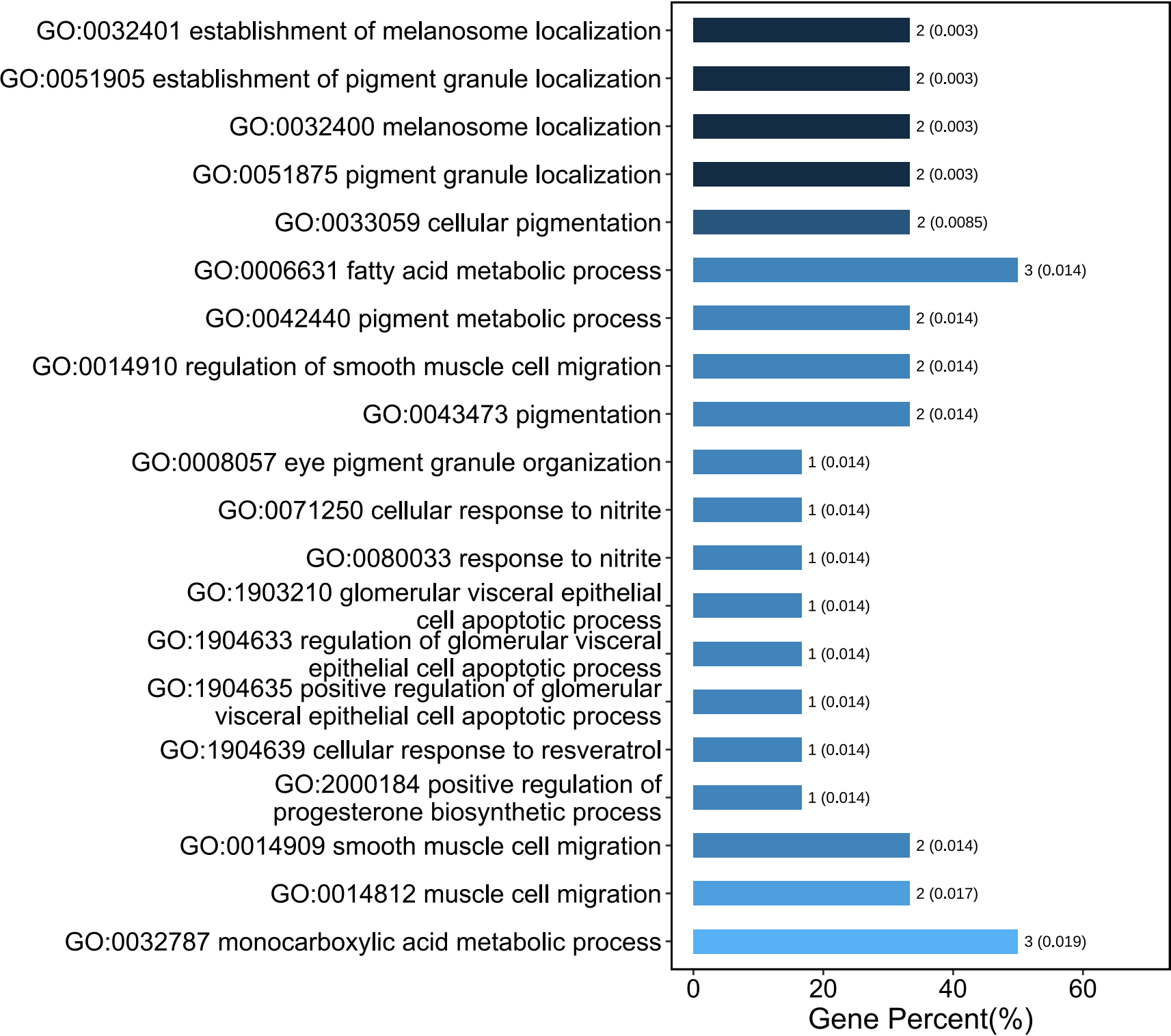

Supplement: Supplementary file 8 [file DataSheet2.ZIP › Gene Ontology (GO) analysis of the downregulated genes in each cluster/Cluster_CDIC-B.P.barplot.pdf]

# Top 20 of GO Enrichment

GOterm

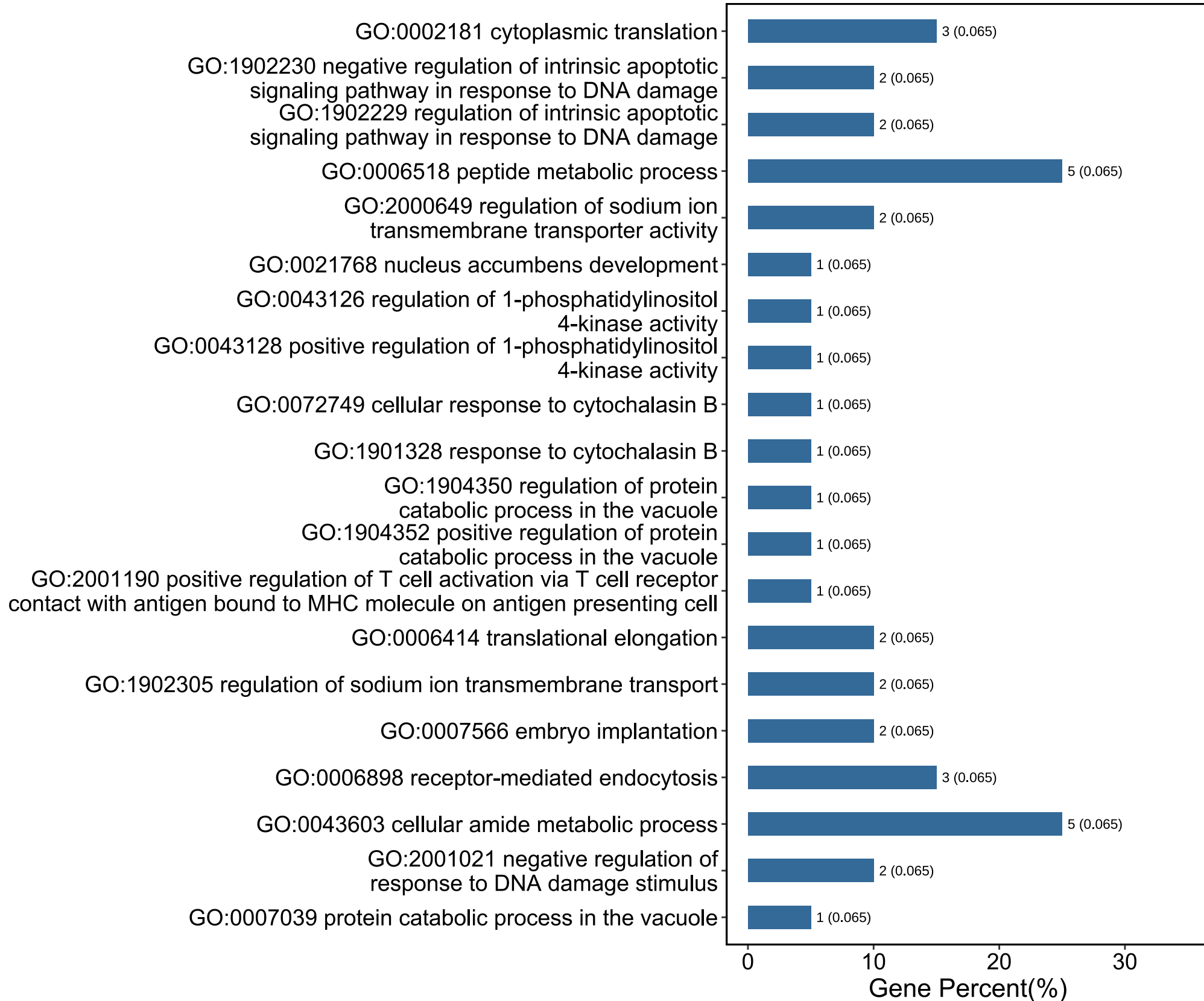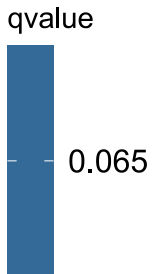

Supplement: Supplementary file 8 [file DataSheet2.ZIP › Gene Ontology (GO) analysis of the downregulated genes in each cluster/Cluster_CDPC.P.barplot.pdf]

# Top 20 of GO Enrichment

GOterm

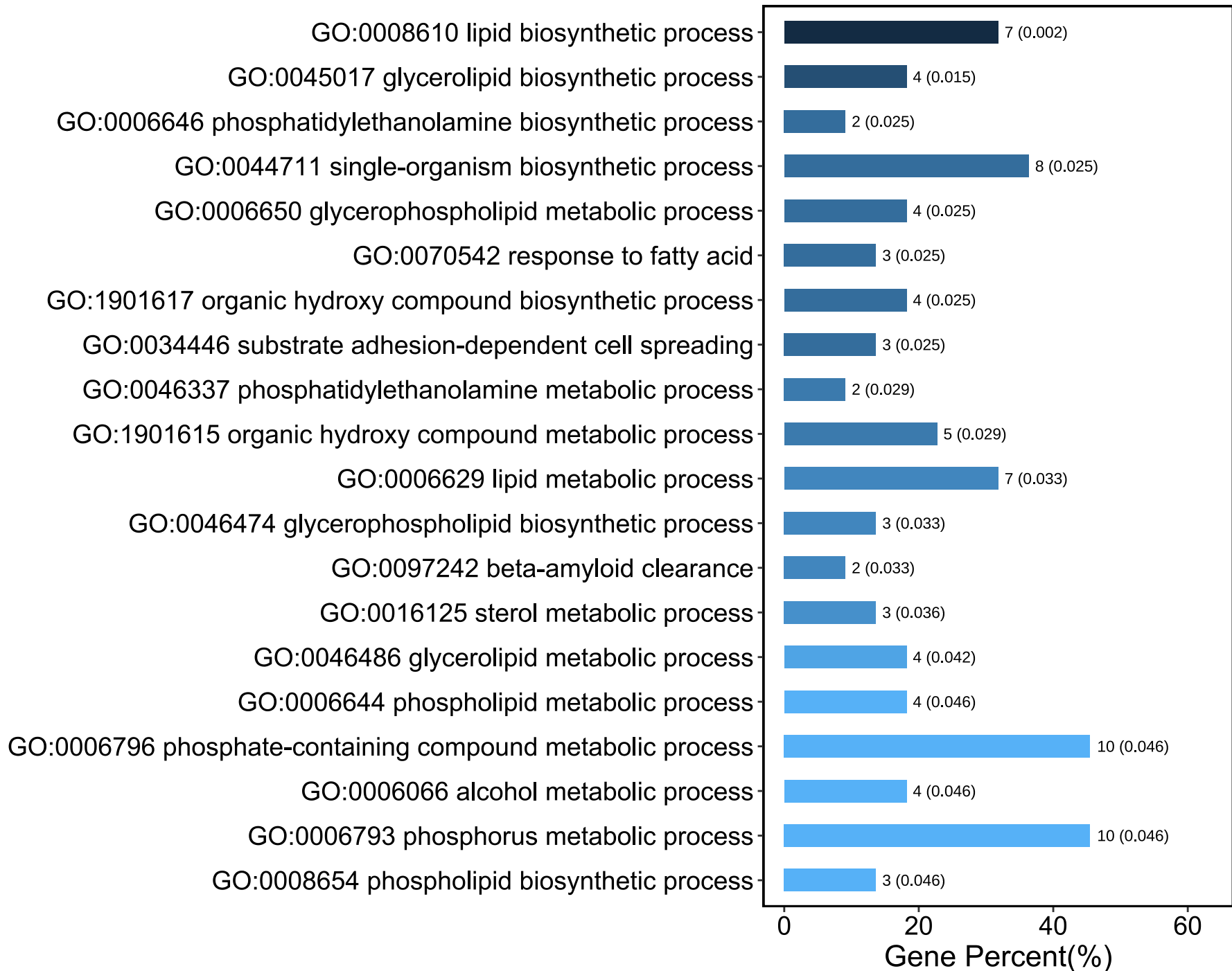

Supplement: Supplementary file 8 [file DataSheet2.ZIP › Gene Ontology (GO) analysis of the downregulated genes in each cluster/Cluster_DCT1.P.barplot.pdf]

# Top 20 of GO Enrichment

GOterm

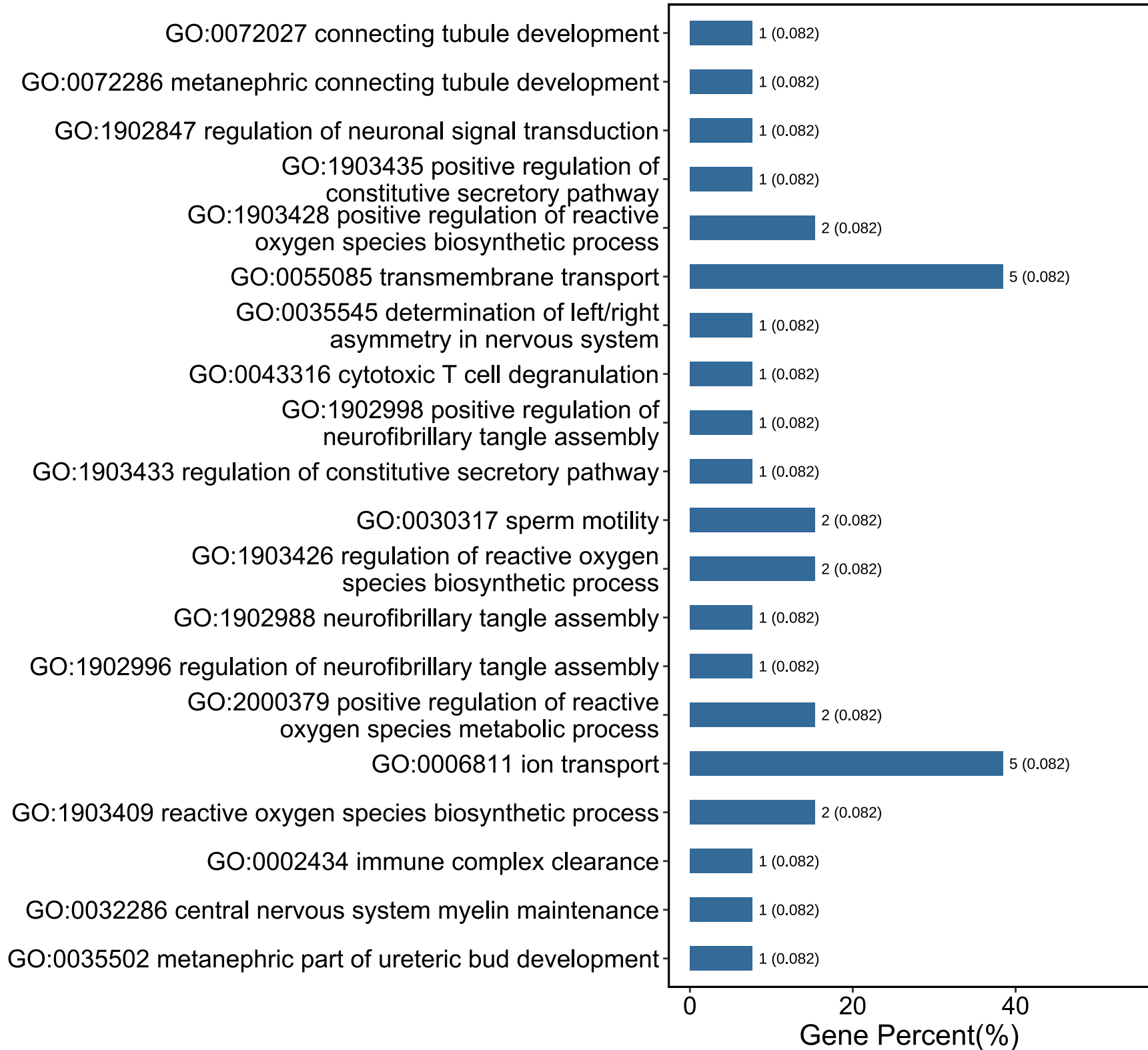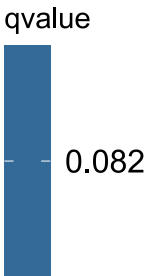

Supplement: Supplementary file 8 [file DataSheet2.ZIP › Gene Ontology (GO) analysis of the downregulated genes in each cluster/Cluster_DCT2.P.barplot.pdf]

# Top 20 of GO Enrichment

GOterm

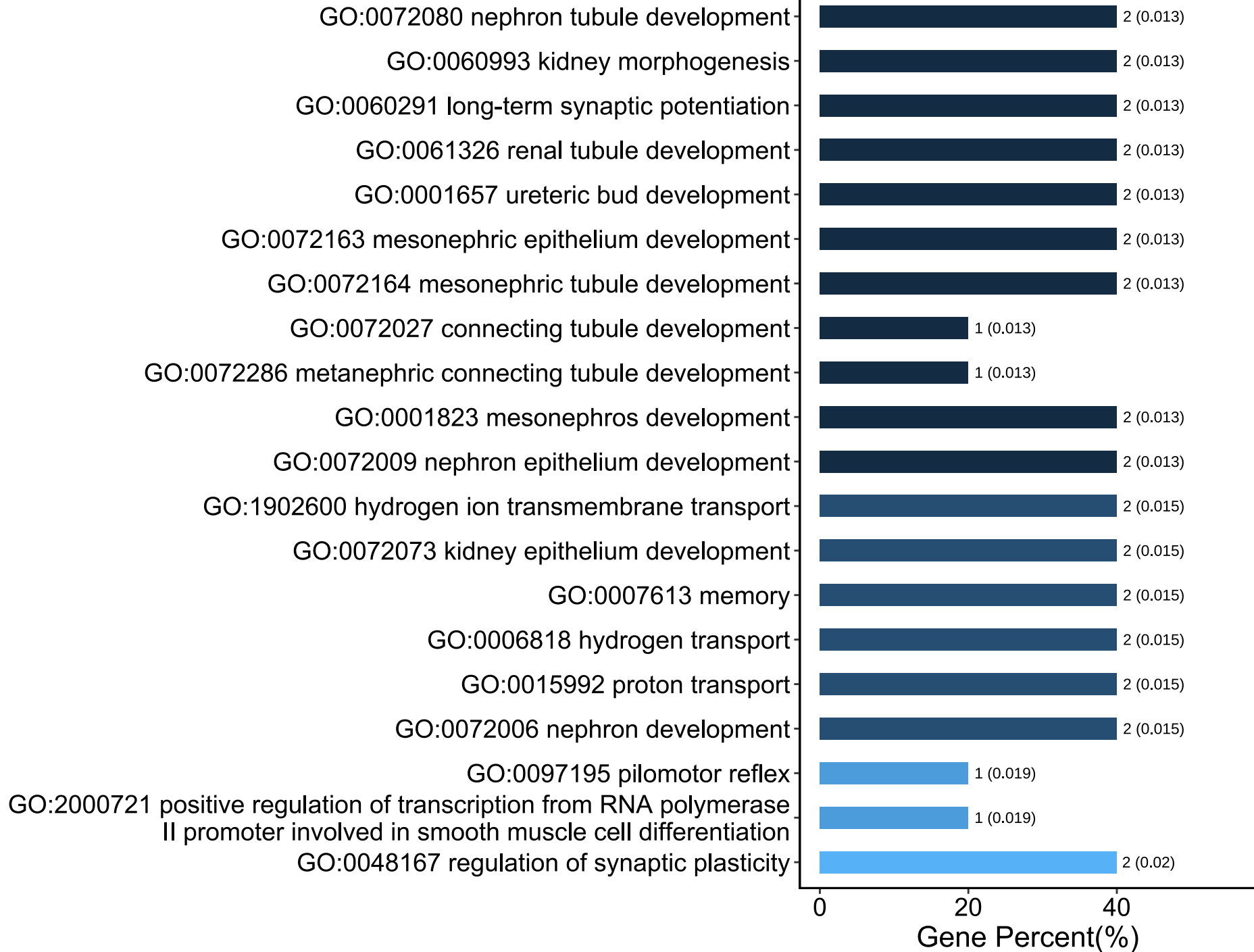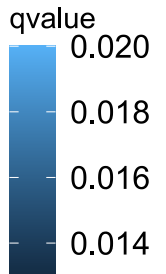

Supplement: Supplementary file 8 [file DataSheet2.ZIP › Gene Ontology (GO) analysis of the downregulated genes in each cluster/Cluster_DCT3.P.barplot.pdf]

# Top 20 of GO Enrichment

GOterm

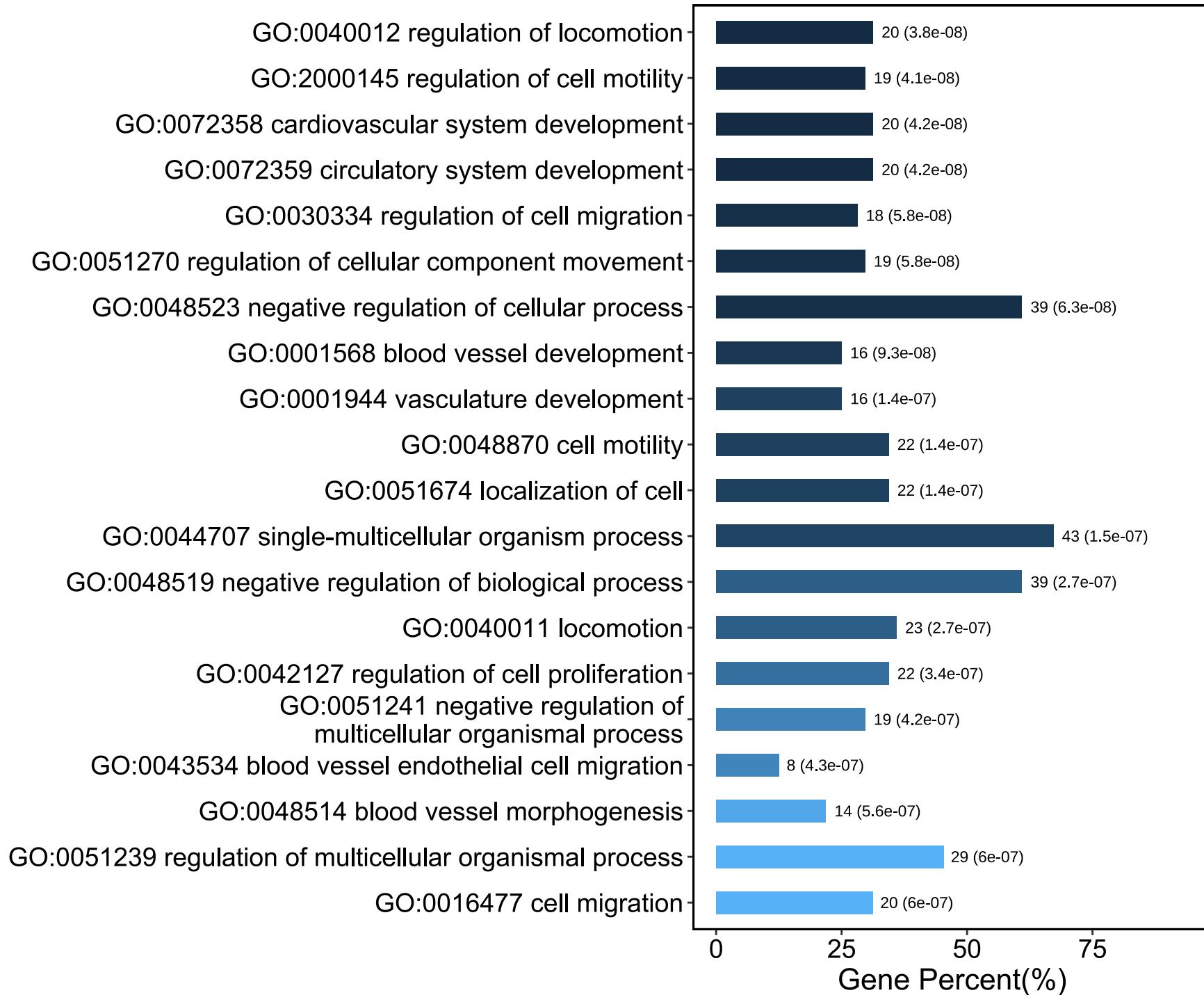

Supplement: Supplementary file 8 [file DataSheet2.ZIP › Gene Ontology (GO) analysis of the downregulated genes in each cluster/Cluster_ENDO.P.barplot.pdf]

# Top 20 of GO Enrichment

GOterm

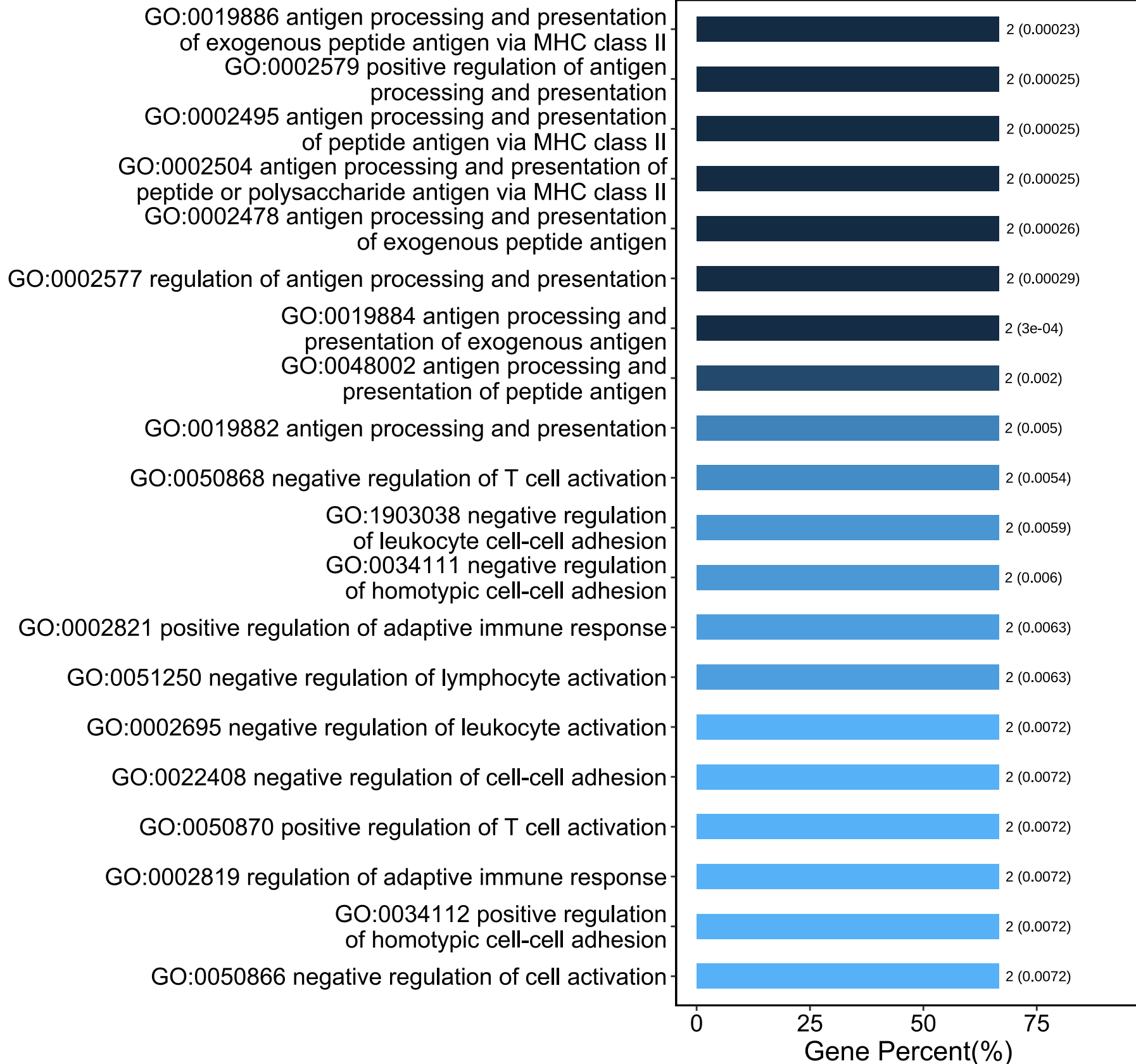

Supplement: Supplementary file 8 [file DataSheet2.ZIP › Gene Ontology (GO) analysis of the downregulated genes in each cluster/Cluster_MACRO.P.barplot.pdf]

# Top 20 of GO Enrichment

GOterm

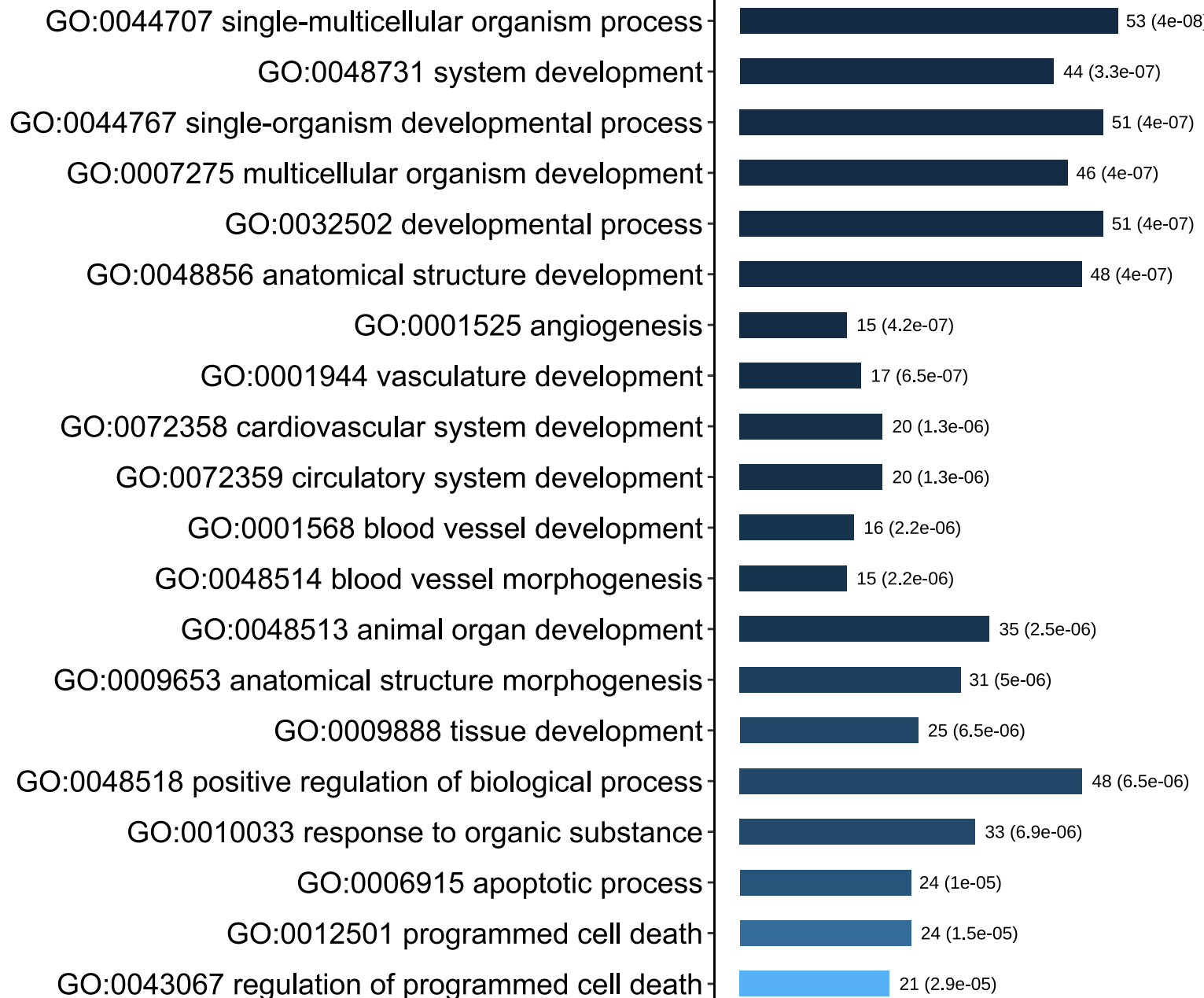

qvalue

2e-05

1e-05

Gene Percent(%)

Supplement: Supplementary file 8 [file DataSheet2.ZIP › Gene Ontology (GO) analysis of the downregulated genes in each cluster/Cluster_MES.P.barplot.pdf]

# Top 20 of GO Enrichment

GOterm

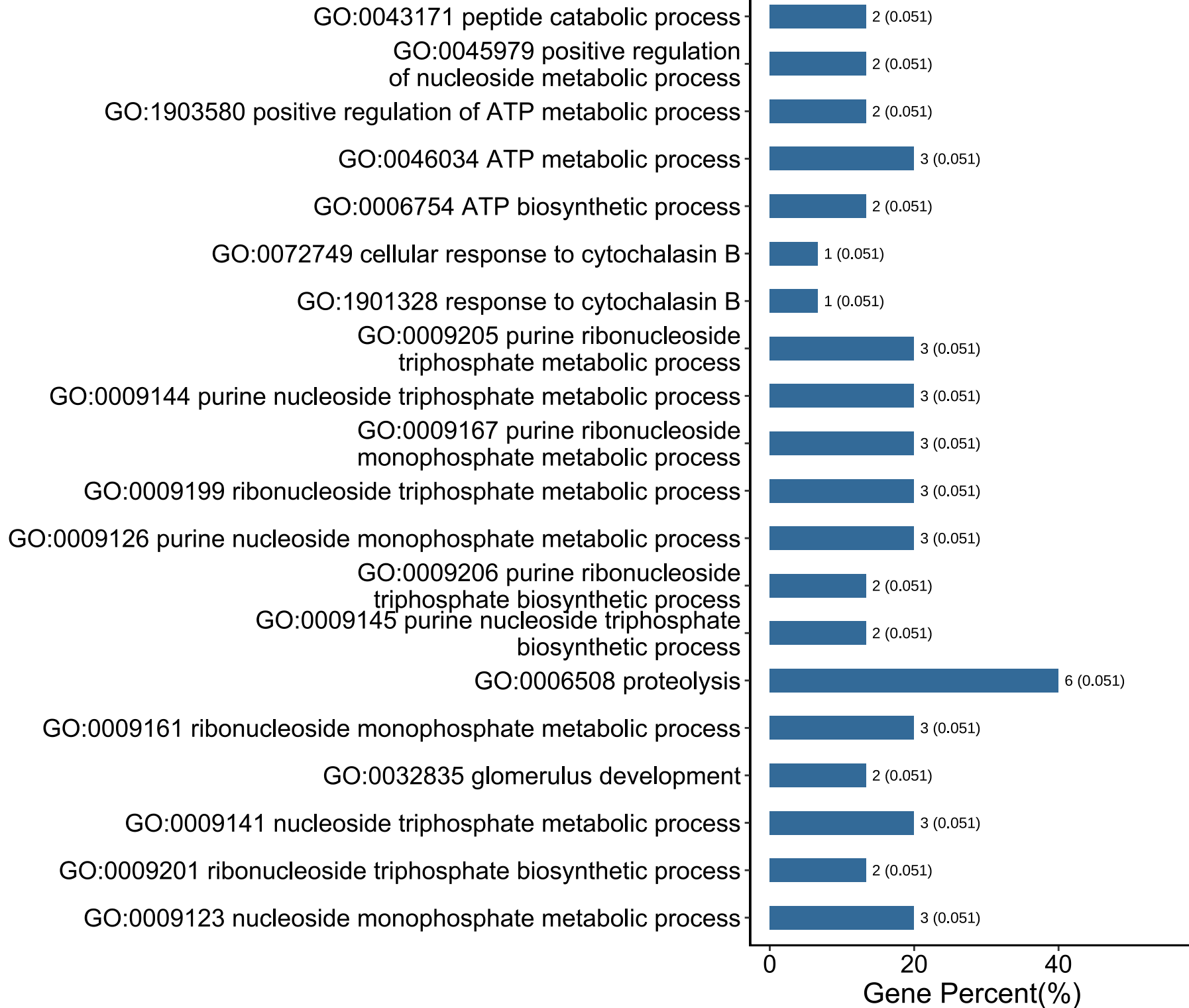

Supplement: Supplementary file 8 [file DataSheet2.ZIP › Gene Ontology (GO) analysis of the downregulated genes in each cluster/Cluster_PODO.P.barplot.pdf]

# Top 20 of GO Enrichment

GOterm

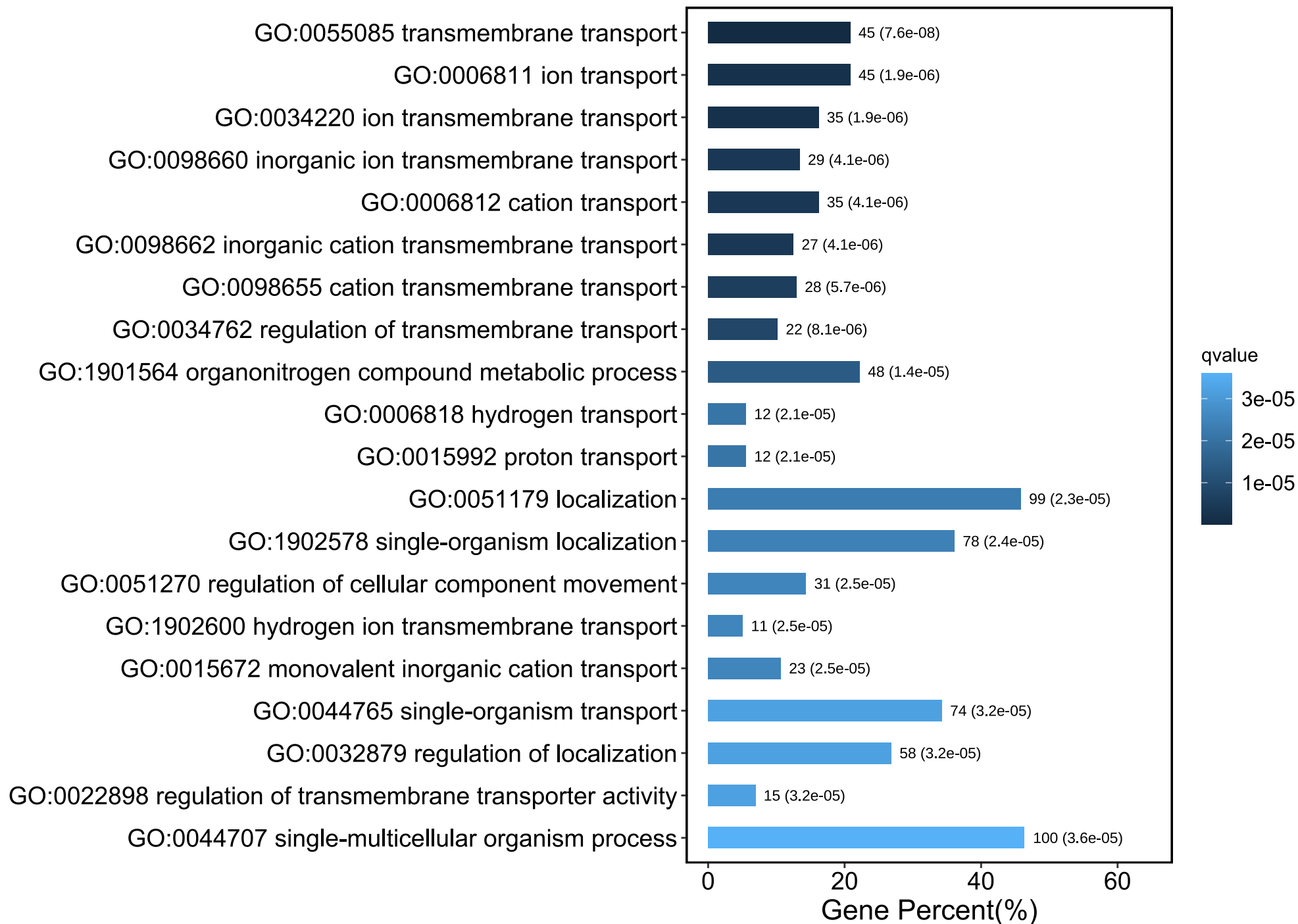

Supplement: Supplementary file 8 [file DataSheet2.ZIP › Gene Ontology (GO) analysis of the downregulated genes in each cluster/Cluster_PT.P.barplot.pdf]

# Top 20 of GO Enrichment

GOterm

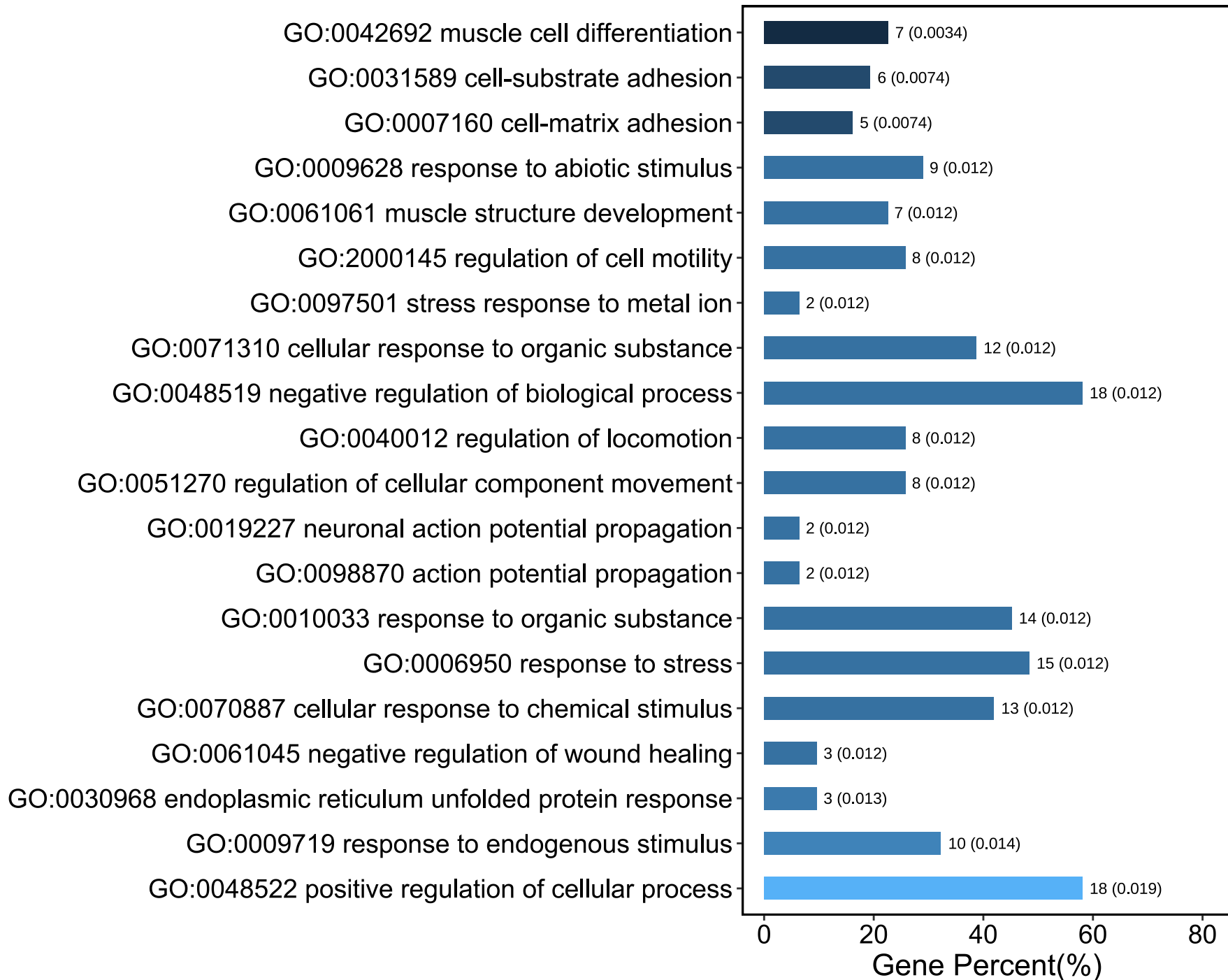

Supplement: Supplementary file 8 [file DataSheet2.ZIP › Gene Ontology (GO) analysis of the downregulated genes in each cluster/Cluster_TAL.P.barplot.pdf]

# Top 20 of GO Enrichment

GOterm

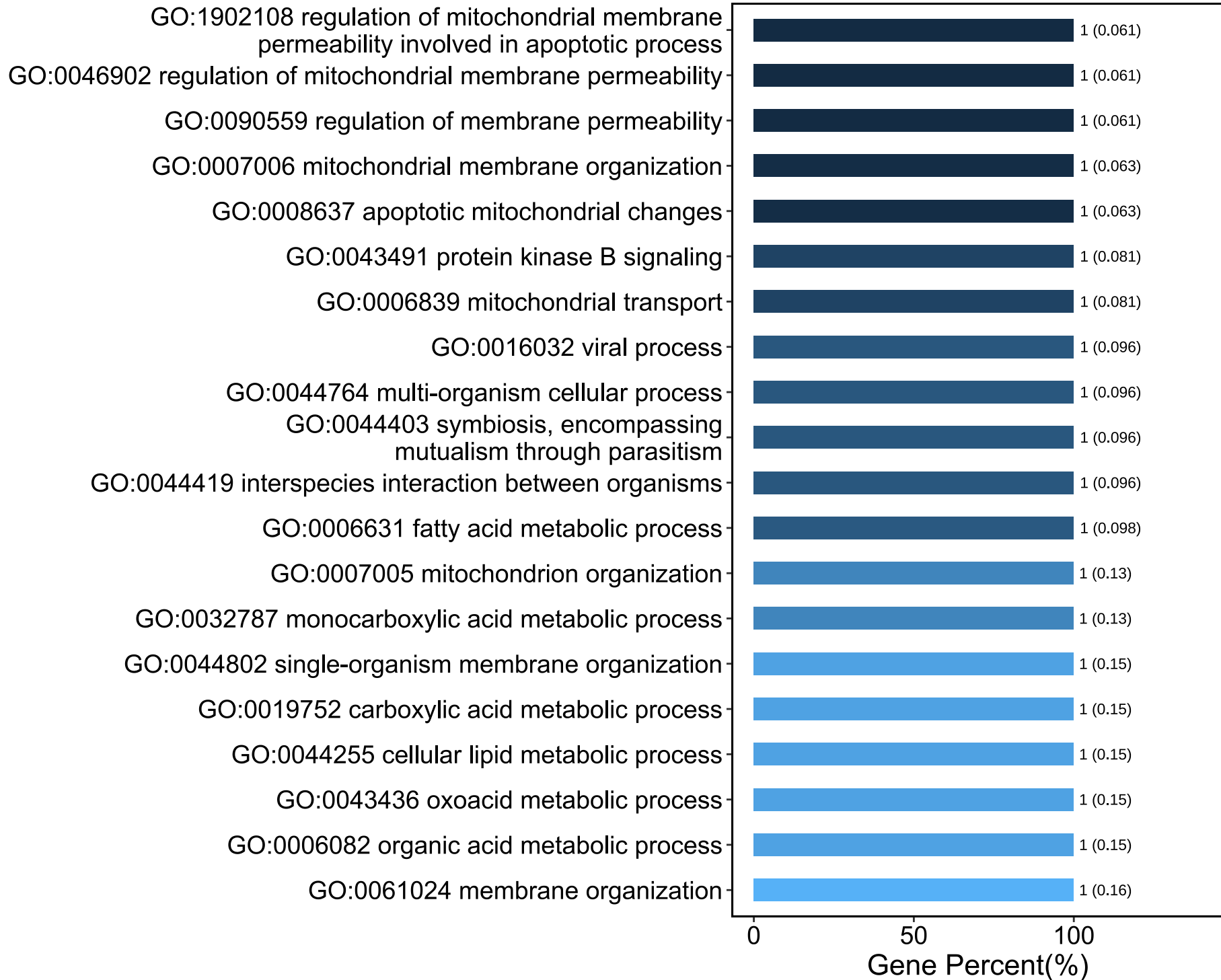

Supplement: Supplementary file 8 [file DataSheet2.ZIP › Gene Ontology (GO) analysis of the downregulated genes in each cluster/Cluster_TEPI.P.barplot.pdf]
