# Supplementary material for: Identification of renal cyst cells of type I Nephronophthisis by single-nucleus RNA sequencing
Source: Front Cell Dev Biol. 2023 Jul 31;11:1192935. doi: 10.3389/fcell.2023.1192935 (PMC10423821; doi:10.3389/fcell.2023.1192935)
Supplement: Supplementary file 2 [file Table1.DOCX]

Table S1 Differential gene analysis of cyst cells and other DCT3

| Gene | Other DCT3 | CYST | Log_2_FC | P value |
| --- | --- | --- | --- | --- |
| Pde10a | 1.155 | 3.177 | 1.460 | 3.84E-08 |
| Tmtc1 | 2.968 | 6.481 | 1.126 | 4.33E-12 |
| Fam129a | 1.389 | 2.889 | 1.056 | 2.09E-07 |
| Zbtb16 | 1.489 | 2.999 | 1.010 | 1.61E-09 |
| Gm15810 | 1.937 | 3.552 | 0.875 | 6.35E-08 |
| Pappa | 4.644 | 8.068 | 0.797 | 1.82E-10 |
| Ube2h | 4.354 | 7.083 | 0.702 | 4.47E-10 |
| Gm37376 | 2.372 | 3.848 | 0.698 | 6.02E-07 |
| Mast4 | 10.865 | 16.098 | 0.567 | 2.15E-11 |
| Tmem117 | 10.085 | 14.641 | 0.538 | 1.02E-09 |
| Nedd4l | 15.969 | 22.680 | 0.506 | 2.17E-11 |
| Tbck | 25.061 | 34.704 | 0.470 | 1.43E-11 |
| Ptprd | 13.714 | 18.566 | 0.437 | 2.16E-07 |
| Naaladl2 | 8.886 | 11.462 | 0.367 | 2.73E-07 |
| Kl | 11.141 | 6.193 | -0.847 | 3.50E-12 |
| Vdr | 4.151 | 2.236 | -0.893 | 1.67E-07 |
| Slc8a1 | 202.796 | 93.734 | -1.113 | 3.61E-09 |
