# Supplementary material for: Identification of renal cyst cells of type I Nephronophthisis by single-nucleus RNA sequencing
Source: Front Cell Dev Biol. 2023 Jul 31;11:1192935. doi: 10.3389/fcell.2023.1192935 (PMC10423821; doi:10.3389/fcell.2023.1192935)
Supplement: Supplementary file 7 [file Table2.DOCX]

Table S2 Differential gene analysis of cyst cells and all other DCT

| Gene | All other | CYST | Log_2_FC | P value |
| --- | --- | --- | --- | --- |
| Mcoln3 | 0.073 | 0.768 | 3.399 | 2.38E-17 |
| Kcnh5 | 0.156 | 1.525 | 3.285 | 4.72E-25 |
| Tmtc1 | 0.787 | 6.481 | 3.041 | 2.04E-74 |
| Col26a1 | 0.469 | 3.462 | 2.884 | 1.05E-59 |
| Spock3 | 0.264 | 1.949 | 2.884 | 6.49E-24 |
| Abca8b | 0.078 | 0.578 | 2.883 | 2.58E-14 |
| Pde1c | 0.438 | 3.197 | 2.867 | 1.40E-42 |
| Kcnc2 | 0.679 | 4.806 | 2.824 | 1.54E-58 |
| Prr5l | 0.244 | 1.724 | 2.818 | 8.08E-34 |
| Aqp3 | 0.605 | 4.172 | 2.786 | 1.69E-62 |
| Gm10848 | 0.125 | 0.856 | 2.781 | 1.83E-22 |
| Gm13269 | 0.159 | 1.081 | 2.769 | 1.43E-18 |
| Gm50368 | 0.111 | 0.756 | 2.765 | 1.68E-13 |
| Acer2 | 0.138 | 0.937 | 2.763 | 6.70E-19 |
| Plcd3 | 0.448 | 3.016 | 2.752 | 1.84E-50 |
| Fanca | 0.311 | 2.074 | 2.737 | 2.98E-38 |
| Rbfox1 | 1.456 | 9.418 | 2.694 | 2.64E-65 |
| Rasgrf2 | 0.179 | 1.126 | 2.655 | 1.57E-23 |
| Tmem150c | 0.193 | 1.210 | 2.646 | 2.52E-23 |
| Prkcq | 0.167 | 1.042 | 2.645 | 1.27E-21 |
| Adh1 | 0.121 | 0.749 | 2.627 | 5.60E-14 |
| Hdac9 | 0.421 | 2.593 | 2.623 | 1.20E-25 |
| Kcne1 | 0.378 | 2.262 | 2.579 | 4.00E-35 |
| Ehf | 0.445 | 2.640 | 2.570 | 4.70E-42 |
| Ptprm | 0.598 | 3.526 | 2.559 | 1.15E-53 |
| Coro2a | 0.110 | 0.647 | 2.552 | 1.71E-12 |
| Mylk | 0.314 | 1.826 | 2.541 | 1.38E-26 |
| Cacnb2 | 1.581 | 9.068 | 2.520 | 1.36E-87 |
| Kif26b | 1.316 | 7.539 | 2.518 | 1.12E-86 |
| Crybg1 | 1.769 | 10.130 | 2.517 | 9.34E-91 |
| Dapp1 | 0.093 | 0.531 | 2.512 | 4.18E-09 |
| Gm29811 | 0.090 | 0.516 | 2.511 | 2.53E-08 |
| Bmpr1b | 2.163 | 12.213 | 2.497 | 5.93E-97 |
| Chst11 | 0.193 | 1.081 | 2.488 | 6.37E-18 |
| Prkch | 0.410 | 2.284 | 2.478 | 9.45E-40 |
| Rhbdl3 | 0.091 | 0.504 | 2.464 | 7.23E-12 |
| Fxyd4 | 0.311 | 1.700 | 2.449 | 1.64E-25 |
| Ackr3 | 0.179 | 0.972 | 2.437 | 4.49E-19 |
| St6gal1 | 0.697 | 3.767 | 2.434 | 1.77E-57 |
| Aqp2 | 2.080 | 11.161 | 2.424 | 1.94E-82 |
| Frmpd4 | 5.445 | 29.103 | 2.418 | 2.75E-88 |
| Tmem229a | 0.190 | 1.010 | 2.414 | 2.80E-19 |
| Samd12 | 0.198 | 1.050 | 2.410 | 1.94E-10 |
| Gpc5 | 3.532 | 18.748 | 2.408 | 3.43E-86 |
| Igf2bp2 | 0.148 | 0.781 | 2.401 | 1.14E-12 |
| Cyp7b1 | 0.353 | 1.846 | 2.386 | 1.59E-23 |
| Fstl1 | 0.114 | 0.593 | 2.385 | 3.87E-09 |
| Slc9a4 | 0.122 | 0.634 | 2.381 | 5.02E-11 |
| Gm45886 | 0.171 | 0.884 | 2.369 | 3.51E-17 |
| Ahnak | 0.371 | 1.911 | 2.364 | 5.08E-31 |
| Grip1 | 2.036 | 10.456 | 2.360 | 1.05E-64 |
| Frem2 | 0.316 | 1.602 | 2.344 | 9.69E-18 |
| Gm15083 | 0.117 | 0.593 | 2.337 | 4.97E-10 |
| Tbck | 6.873 | 34.704 | 2.336 | 3.60E-139 |
| Mast4 | 3.223 | 16.098 | 2.320 | 7.52E-113 |
| Spink8 | 0.239 | 1.185 | 2.309 | 8.05E-19 |
| Cav1 | 0.975 | 4.781 | 2.295 | 7.86E-50 |
| Gm9403 | 0.098 | 0.477 | 2.287 | 1.95E-10 |
| Plppr1 | 1.607 | 7.826 | 2.284 | 6.46E-68 |
| Cers4 | 0.119 | 0.565 | 2.252 | 3.30E-09 |
| Gm20713 | 0.408 | 1.942 | 2.249 | 3.45E-25 |
| Phkg1 | 0.160 | 0.728 | 2.184 | 2.15E-10 |
| Abr | 2.679 | 12.004 | 2.164 | 1.47E-82 |
| Fam129a | 0.650 | 2.889 | 2.152 | 4.81E-31 |
| Myh10 | 0.530 | 2.333 | 2.137 | 2.53E-32 |
| Prrg1 | 0.298 | 1.287 | 2.111 | 9.37E-15 |
| Scin | 0.472 | 2.035 | 2.109 | 8.60E-23 |
| Nav1 | 0.403 | 1.718 | 2.092 | 1.33E-20 |
| Lingo2 | 0.203 | 0.864 | 2.088 | 1.46E-07 |
| Unc5c | 0.129 | 0.547 | 2.082 | 3.36E-08 |
| Glb1l2 | 0.196 | 0.819 | 2.064 | 9.52E-11 |
| Sgk1 | 0.418 | 1.737 | 2.056 | 9.93E-21 |
| Dpp4 | 0.253 | 1.030 | 2.023 | 1.96E-13 |
| Pde8b | 1.189 | 4.824 | 2.020 | 4.18E-45 |
| Car2 | 0.332 | 1.345 | 2.017 | 1.53E-17 |
| Apela | 0.199 | 0.804 | 2.016 | 1.11E-11 |
| 4930426D05Rik | 0.132 | 0.531 | 2.009 | 9.75E-08 |
| Smim6 | 0.169 | 0.671 | 1.993 | 4.29E-08 |
| Gm15581 | 0.650 | 2.583 | 1.991 | 6.22E-26 |
| Hsd11b2 | 3.133 | 12.378 | 1.982 | 3.61E-88 |
| Naaladl2 | 2.904 | 11.462 | 1.981 | 3.52E-82 |
| Camkk2 | 0.425 | 1.671 | 1.976 | 1.03E-18 |
| Lamc2 | 0.359 | 1.406 | 1.971 | 4.71E-18 |
| Fgf12 | 0.378 | 1.473 | 1.962 | 1.64E-12 |
| Tgfbr2 | 0.345 | 1.314 | 1.930 | 1.99E-14 |
| Ces1d | 0.222 | 0.842 | 1.924 | 7.31E-09 |
| Srcin1 | 0.450 | 1.706 | 1.923 | 6.39E-22 |
| Mfhas1 | 0.526 | 1.988 | 1.918 | 1.10E-22 |
| Gm15283 | 1.397 | 5.233 | 1.905 | 6.63E-38 |
| Fyn | 0.207 | 0.762 | 1.882 | 3.17E-09 |
| Tpd52l1 | 0.728 | 2.679 | 1.879 | 4.84E-27 |
| Dop1b | 0.790 | 2.894 | 1.874 | 6.20E-30 |
| Plxdc2 | 1.673 | 6.041 | 1.853 | 6.12E-46 |
| Atp2b2 | 1.360 | 4.902 | 1.850 | 3.94E-45 |
| Tmem178 | 2.106 | 7.571 | 1.846 | 1.71E-40 |
| Ptger1 | 0.146 | 0.524 | 1.842 | 8.34E-07 |
| Ephb2 | 0.341 | 1.221 | 1.840 | 1.42E-14 |
| Veph1 | 1.486 | 5.282 | 1.830 | 1.21E-40 |
| Il1r1 | 0.220 | 0.782 | 1.828 | 4.30E-08 |
| Lypd6 | 0.395 | 1.402 | 1.826 | 5.17E-14 |
| Pfkfb3 | 0.315 | 1.116 | 1.825 | 3.33E-11 |
| Tnfaip8 | 0.258 | 0.915 | 1.825 | 7.49E-11 |
| Tpm1 | 0.235 | 0.825 | 1.814 | 3.48E-09 |
| Tmem45b | 1.763 | 6.193 | 1.813 | 5.41E-44 |
| Sgcd | 0.499 | 1.749 | 1.810 | 8.51E-16 |
| Pappa | 2.309 | 8.068 | 1.805 | 7.13E-50 |
| Slc4a8 | 0.173 | 0.605 | 1.804 | 5.44E-07 |
| Mcoln4 | 0.961 | 3.470 | 1.686 | 5.47E-08 |
| Kcnh6 | 0.965 | 3.466 | 1.675 | 5.54E-08 |
| Tmtc2 | 0.968 | 3.463 | 1.663 | 5.61E-08 |
