# Supplementary material for: Identification of renal cyst cells of type I Nephronophthisis by single-nucleus RNA sequencing
Source: Front Cell Dev Biol. 2023 Jul 31;11:1192935. doi: 10.3389/fcell.2023.1192935 (PMC10423821; doi:10.3389/fcell.2023.1192935)
Supplement: Supplementary file 8 [file DataSheet2.ZIP › Gene Ontology (GO) analysis of the downregulated genes in each cluster/Cluster_DTL.P.barplot.pdf]

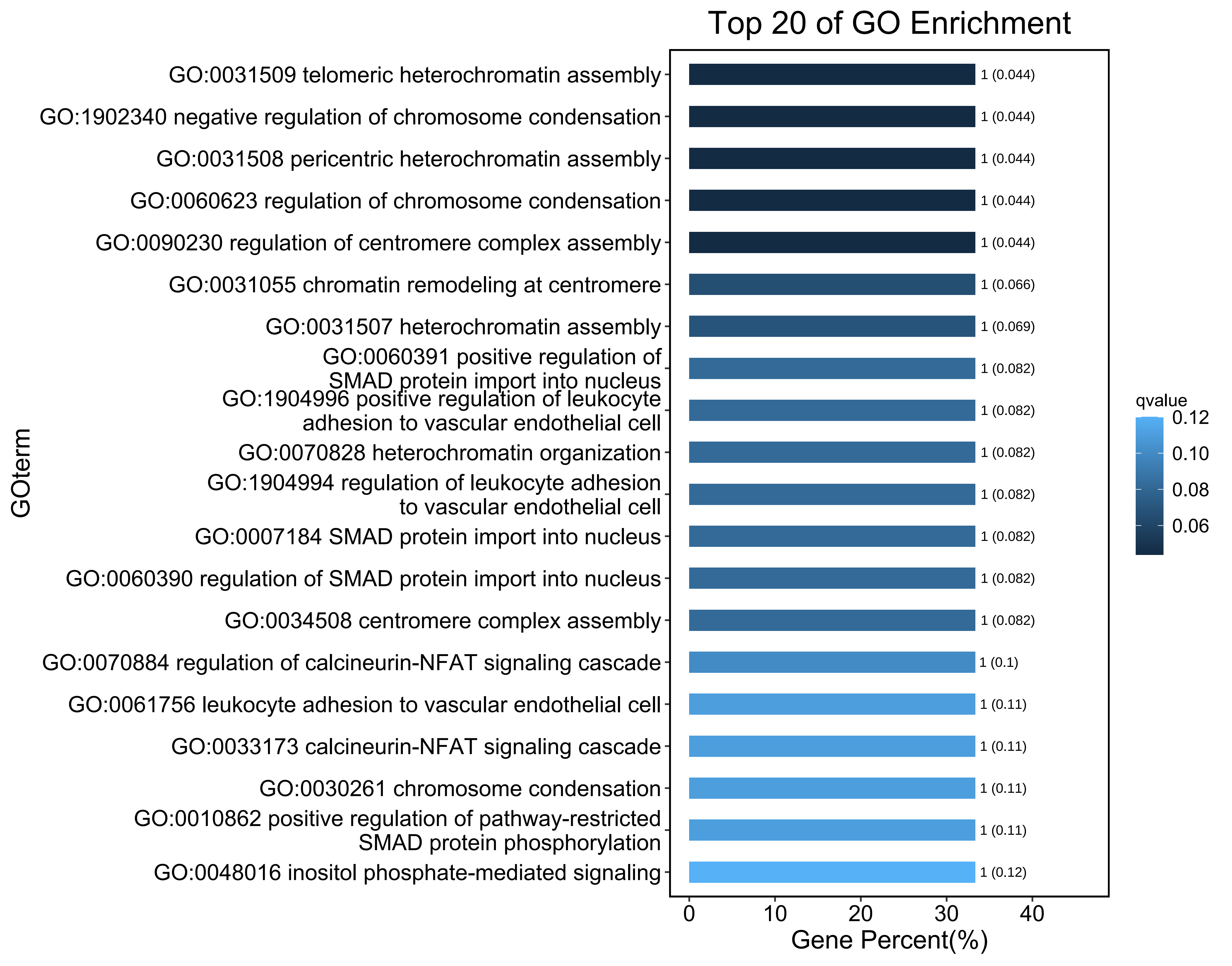
Top 20 of GO Enrichment
GO:0031509 telomeric heterochromatin assembly
GO:1902340 negative regulation of chromosome condensation
GO:0031508 pericentric heterochromatin assembly
GO:0060623 regulation of chromosome condensation
GO:0090230 regulation of centromere complex assembly
GO:0031055 chromatin remodeling at centromere
GO:0031507 heterochromatin assembly
GO:0060391 positive regulation of SMAD protein import into nucleus
GO:1904996 positive regulation of leukocyte adhesion to vascular endothelial cell
GO:0070828 heterochromatin organization
GO:1904994 regulation of leukocyte adhesion to vascular endothelial cell
GO:0007184 SMAD protein import into nucleus
GO:0060390 regulation of SMAD protein import into nucleus
GO:0034508 centromere complex assembly
GO:0070884 regulation of calcineurin-NFAT signaling cascade
GO:0061756 leukocyte adhesion to vascular endothelial cell
GO:0033173 calcineurin-NFAT signaling cascade
GO:0030261 chromosome condensation
GO:0010862 positive regulation of pathway-restricted SMAD protein phosphorylation
GO:0048016 inositol phosphate-mediated signaling
1 (0.044)
1 (0.044)
1 (0.044)
1 (0.044)
1 (0.044)
1 (0.066)
1 (0.069)
1 (0.082)
1 (0.082)
1 (0.082)
1 (0.082)
1 (0.082)
1 (0.082)
1 (0.082)
1 (0.1)
1 (0.11)
1 (0.11)
1 (0.11)
1 (0.11)
1 (0.12)
GOterm
0
10
20
30
40
Gene Percent(%)
qvalue
0.12
0.10
0.08
0.06
