# Supplementary material for: Identification of renal cyst cells of type I Nephronophthisis by single-nucleus RNA sequencing
Source: Front Cell Dev Biol. 2023 Jul 31;11:1192935. doi: 10.3389/fcell.2023.1192935 (PMC10423821; doi:10.3389/fcell.2023.1192935)
Supplement: Supplementary file 9 [file DataSheet1.docx]

**Supplementary methods**

***Data quality control and gene expression quantification***

10X Genomics Cell Ranger software (version 3.1.0) was used to convert raw BCL files to FASTQ files and for alignment and count quantification.

*Data quality control and genome alignment*

Data quantification was performed using Cell Ranger. The Illumina double-end sequencing results contain Read1 and Read2. Read1 contains a 16 bp GemCode barcode (to distinguish between different cells) and a 10 bp UMI (Unique Molecular Identifier, to distinguish between different RNA molecules; an mRNA molecule will be labeled by a UMI); Read2 is a sequence fragment of cDNA.

Cell Ranger uses an aligner called STAR (https://github.com/alexdobin/STAR), which performs splicing-aware alignment of Reads2 to the genome. Cell Ranger further aligns exonic reads back to annotated transcripts, and only uni-mapped reads can be used for UMI counting. Cell Ranger will continue to filter and correct barcodes and UMIs. Only the filtered and corrected barcodes and UMI reads can be used for UMI counting.

*Gene expression quantification*

Before quantification, the UMI sequences were corrected for sequencing errors, and valid barcodes were identified based on the EmptyDrops method(Lun et al., 2019). The cell-by-gene matrices were produced via UMI counting and cell barcode calling.

***Cell clustering***

The cell-by-gene matrices for each sample were individually imported into Seurat(Butler et al., 2018) version 3.1.1 for downstream analysis.

*Expression QC*

Cells with an unusually high number of UMIs (≥8000) or mitochondrial gene percentage (≥10%) and cells with fewer than 500 or more than 4000 genes were filtered out. Additionally, doublet GEMs were also filtered out by using the tool DoubletFinder (v2.0.3), which generates artificial doublets, uses the PC distance to find each cell’s proportion of artificial k nearest neighbors (pANN) and then ranks them according to the expected number of doublets(McGinnis et al., 2019).

*Normalizing the data*

After removing unwanted cells from the dataset, we employed a global-scaling normalization method, “LogNormalize”, that normalizes the gene expression measurements for each cell by the total expression, multiplies this by a scale factor (10,000 by default), and log-transforms the results. The formula is shown as follows: A gene expression level = log [1 + (UMI_A_/UMI_Total_) × 10000]

*Batch effect correction*

We used Harmony to minimize batch effects and behavioral condition effects on clustering(Korsunsky et al., 2019). The Harmony algorithm inputs a PCA embedding of cells, along with their batch assignments, and returns a batch corrected embedding.

*Principal component* analysis (PCA)

The integrated expression matrix was scaled and performed on principal component analysis for dimensional reduction. We randomly permuted a subset of the data (1% by default) and reran the PCA, constructing a ‘null distribution’ of gene scores, and repeated this procedure. We identified ‘significant’ PCs as those with a strong enrichment of low p value genes for downstream clustering and dimensional reduction(Chung and Storey, 2015).

*Clustering cells*

Seurat implements a graph-based clustering approach. Distances between cells were calculated based on previously identified PCs. Briefly, Seurat embeds cells in a shared-nearest neighbor (SNN) graph, with edges drawn between cells via similar gene expression patterns. To partition this graph into highly interconnected quasi-cliques or communities, we first constructed the SNN graph based on the Euclidean distance in PCA space and refined the edge weights between any two cells based on the shared overlap in their local neighborhoods (Jaccard distance). We then clustered the cells using the Louvain(Rotta and Noack, 2011) method to maximize modularity.

*t-SNE (t-distributed stochastic neighbor embedding) visualization*

For visualization of clusters, t-distributed stochastic neighbor embedding (t-SNE) was generated using the same PCs(Zhou and Jin, 2020).

***Differentially expressed gene (upregulation) analysis and cell type annotation***

The expression value of each gene in a given cluster was compared to those in the rest of the cells using the Wilcoxon rank sum test(Camp et al., 2017). Significantly upregulated genes were identified using a number of criteria. First, genes had to be at least 1.28-fold overexpressed in the target cluster. Second, genes had to be expressed in more than 25% of the cells belonging to the target cluster. Third, the p value had to be less than 0.05. We generated cluster-specific marker genes by performing upregulated gene expression analysis and performed cell type annotation based on matching cluster-specific marker genes to known cell type-specific markers.

***Differentially expressed gene analysis for groups***

Seurat was used for differentially expressed gene analysis. New idents were set up for analysis as “group_cluster” or “group_cell type”(Stuart et al., 2019). Then, we used a hurdle model in MAST (Model-based Analysis of Single-cell Transcriptomics)(Finak et al., 2015) to find differentially expressed genes for a group in one cluster. We identified differentially expressed genes as follows: ① |logFC|≥1; ② p_value_adj≤0.05; ③ the percentage of cells in a specific cluster in which the gene was detected was more than 10%.

***Reconstructing differentiation trajectories by Monocle2***

The single-cell trajectory was analyzed using a matrix of cells and gene expression by Monocle(Trapnell et al., 2014) (Version 2.10.1). Monocle reduced the space to two dimensions and ordered the cells (sigma = 0.001, lambda = NULL, param.gamma = 10, tol = 0.001)(Qiu et al., 2017). Once the cells were ordered, we could visualize the trajectory in the reduced dimensional space. The trajectory has a tree-like structure, including tips and branches.

***Kidney histopathology and immunohistochemistry***

The kidneys were collected and immediately fixed with 4% paraformaldehyde at 4 °C overnight. Then, they were treated with an alcohol series and xylene before embedding in low-melting temperature paraffin. Four-micrometer-thick paraffin kidney tissue sections were subjected to deparaffinization and heat-induced antigen retrieval, washed with PBS, and blocked with peroxidase to remove endogenous hydrogen peroxide. Then, the sections were blocked with normal goat serum working solution and incubated with FAM129A (Proteintech, 21333-1-AP, 1:100) and PCNA (Abcam, ab29, 1:1000) antibodies in a humidified chamber at 4 °C overnight. Then, the sections were incubated with secondary antibodies and horseradish enzyme-labeled streptavidin working solution at room temperature for 15 minutes. Finally, the sections were stained with a 3,3’-diaminobenzidine (DAB) kit (ZSGB-BIO, ZLI-9017).

***Immunofluorescence studies***

Kidney tissue sections (4 μm) were subjected to deparaffinization and heat-induced antigen retrieval. After blocking with 10% normal goat serum, the sections were incubated with FAM129A (Proteintech, 21333-1-AP, 1:100) antibodies in a humidified chamber at 4 °C overnight. Then, the sections were incubated with fluorophore-labeled secondary antibodies at room temperature for 1 hour. After washing with PBS, the sections were stained with 4′,6-diamidino-2-phenylindole (DAPI) and mounted in antifade mounting medium, and the fluorescence was detected under a laser-scanning confocal microscope.

***TUNEL assay for apoptosis***

Kidney tissue sections (4 μm) were subjected to deparaffinization. First, 100 µL of 20 µg/mL Proteinase K solution was added dropwise to each sample and incubated for 20 min at room temperature. The samples were washed and incubated with 100 μL TUNEL Equilibration Buffer for 5 min; then, 50 μL TUNEL Reaction Mix (containing 1 μL TdT enzyme) was added, and the samples were incubated for 2 hours at room temperature with protection from light. Samples were then washed three times for 5 minutes each with a buffer of 0.1% Triton X-100 (containing 5 mg/ml BSA) in 1x PBS to reduce background staining. The sections were stained with 4′,6-diamidino-2-phenylindole (DAPI) and mounted in antifade mounting medium, and the fluorescence was detected under a laser-scanning confocal microscope.

**Supplementary References**

1. Butler, A., Hoffman, P., Smibert, P., Papalexi, E., and Satija, R. (2018). Integrating single-cell transcriptomic data across different conditions, technologies, and species. *Nat. Biotechnol.* 36, 411-420. doi:10.1038/nbt.4096
2. Camp, J. G., Sekine, K., Gerber, T., Loeffler-Wirth, H., Binder, H., Gac, M., et al. (2017). Multilineage communication regulates human liver bud development from pluripotency. *Nature* 546, 533-538. doi:10.1038/nature22796
3. Chung, N. C., and Storey, J. D. (2015). Statistical significance of variables driving systematic variation in high-dimensional data. *Bioinformatics* 31, 545-554. doi:10.1093/bioinformatics/btu674
4. Finak, G., McDavid, A., Yajima, M., Deng, J., Gersuk, V., Shalek, A. K., et al. (2015). MAST: a flexible statistical framework for assessing transcriptional changes and characterizing heterogeneity in single-cell RNA sequencing data. *Genome Biol.* 16, 278. doi:10.1186/s13059-015-0844-5
5. Korsunsky, I., Millard, N., Fan, J., Slowikowski, K., Zhang, F., Wei, K., et al. (2019). Fast, sensitive and accurate integration of single-cell data with Harmony. *Nat. Methods* 16, 1289-1296. doi:10.1038/s41592-019-0619-0
6. Lun, A., Riesenfeld, S., Andrews, T., Dao, T. P., Gomes, T., and Marioni, J. C. (2019). EmptyDrops: distinguishing cells from empty droplets in droplet-based single-cell RNA sequencing data. *Genome Biol.* 20, 63. doi:10.1186/s13059-019-1662-y
7. McGinnis, C. S., Murrow, L. M., and Gartner, Z. J. (2019). DoubletFinder: Doublet Detection in Single-Cell RNA Sequencing Data Using Artificial Nearest Neighbors. *Cell Syst* 8, 329-337.e4. doi:10.1016/j.cels.2019.03.003
8. Qiu, X., Mao, Q., Tang, Y., Wang, L., Chawla, R., Pliner, H. A., et al. (2017). Reversed graph embedding resolves complex single-cell trajectories. *Nat. Methods* 14, 979-982. doi:10.1038/nmeth.4402
9. Rotta, R., and Noack, A. (2011). Multilevel local search algorithms for modularity clustering. *Journal of Experimental Algorithmics* 16, 2(3). doi:10.1145/1963190.1970376
10. Stuart, T., Butler, A., Hoffman, P., Hafemeister, C., Papalexi, E., Mauck, W. M. 3rd, et al. (2019). Comprehensive Integration of Single-Cell Data. *Cell* 177, 1888-1902.e21. doi:10.1016/j.cell.2019.05.031
11. Trapnell, C., Cacchiarelli, D., Grimsby, J., Pokharel, P., Li, S., Morse, M., et al. (2014). The dynamics and regulators of cell fate decisions are revealed by pseudotemporal ordering of single cells. *Nat. Biotechnol.* 32, 381-386. doi:10.1038/nbt.2859
12. Zhou, B., and Jin, W. (2020). Visualization of Single Cell RNA-Seq Data Using t-SNE in R. *Methods Mol. Biol.* 2117, 159-167. doi:10.1007/978-1-0716-0301-7_8

**Supplementary figure captions**

**Figure S1. Immunofluorescence staining of Niban1 and THP.**

Nuclei were stained with Niban1 (i, red), THP(ii, green), DAPI (iii, blue) and Merge (iv). (Immunofluorescence, scale bar: 10 μm).

**Figure S2.** **The expression levels of genes associated with proliferation and apoptosis in cyst cells compared to all other renal cells**

The expression levels of PCNA, Cyclin D1 and Caspase3 were all increased in cyst cells compared to all other renal cells. (Horizontal coordinate: PCNA: proliferating cell nuclear antigen, CCND1: cyclin D1, Casp3: caspase3. The vertical coordinate is the relative level of gene expression. All other: all other renal cells, CYST: cyst cells.)

**Figure S3. Cystic cells continue to express marker genes of early development and dedifferentiation**

A: Heatmap of the mean open values of the regulon subpopulation and gene expression levels between cyst cells and all other distal convoluted tubule cells. The regulon activity of each cell was calculated based on the expression of the regulon target gene in each cell; using AUCell, we converted the regulon activity value into an open value, 0 or 1, with a score of 0 indicating that the regulon was closed and a score of 1 indicating that the regulon was open. By calculating the average openness of the regulons in each cell subpopulation, it was possible to explore the openness characteristics of the regulons in each cell subpopulation. The darker the color is, the greater the average open value of the cell subpopulation regulons (each column represents a cell subpopulation, and each row represents a regulon and the number of target genes regulated by this regulon). B: The expression of regulons associated with early development and dedifferentiation**,** such as Pax8, Pax2, and Gata3, were elevated. (horizontal coordinates: gene names; vertical coordinates: relative levels of gene expression). (All other DCT: all distal convoluted tubule cells except cyst cells, CYST: cyst cells.)

**Figure S4. The t-SNE projections of marker genes of macrophage subtypes**

A and B: Comparison of marker genes of macrophage subtypes revealed that the macrophages isolated in our study were both CD68 positive (A) and CD86 positive (B), which indicates that they are M2b-like macrophages (CD68+/CD86+). (The plots are colored according to mean gene expression, blue=high, gray=low.)

**Table S1** **Differential gene analysis of cyst cells and other DCT3**

CYST：Mean expression of the gene in cyst cells

Gene: Gene name

Log_2_FC: Logarithmic value of the mean fold difference in expression of the gene in cyst cells and other DCT3

Other DCT3：Mean expression of the gene in the remaining DCT3 cells

P value: P-value for hypergeometric test

**Table S2** **Differential gene analysis of cyst cells and all other DCT**

All other：Mean expression of the gene in the remaining DCT cells

CYST：Mean expression of the gene in cyst cells

Gene: Gene name

Log_2_FC: Logarithmic value of the mean fold difference in expression of the gene in the remaining DCT cells and cyst cells

P value: P-value for hypergeometric test
